# Estimating the effect of hypothetical dietary protein interventions on changes in body composition of postmenopausal women over 3 years using data from the Women’s Health Initiative (WHI) Study: an emulated target trial

**DOI:** 10.1038/s41366-025-01978-0

**Published:** 2026-01-09

**Authors:** Jiarui Li, Luohua Jiang, Nazmus Saquib, Philippe Jean-Luc Gradidge, Simin Liu, Linda Van Horn, Phyllis A. Richey, David S. Timberlake, Hind A. Beydoun, Longjian Liu, Jie Li, Andrew O. Odegaard

**Affiliations:** 1 Department of Epidemiology and Biostatistics, University of California Irvine, Irvine, CA USA; 2 Center for Global Cardiometabolic Health and Nutrition, University of California Irvine, Irvine, CA USA; 3 Department of Clinical Sciences, Sulaiman Al Rajhi University. Al Bukayriyah, Al-Qassim, Saudi Arabia; 4 Department of Exercise Science and Sports Medicine, School of Therapeutic Sciences, Faculty of Health Sciences, University of the Witwatersrand, Johannesburg, South Africa; 5 Department of Preventive Medicine, Nutrition Division. Northwestern University, Evanston, IL USA; 6 Department of Preventive Medicine, The University of Tennessee Health Science Center, Memphis, TN USA; 7 Department of Population Health & Disease. University of California, Irvine, Irvine, CA USA; 8 Department of Research Programs, Fort Belvoir Community Hospital, Fort Belvoir, VA USA; 9 Department of Epidemiology and Biostatistics, Drexel University, Philadelphia, PA USA; 10 Department of Epidemiology, Brown University, Providence, RI USA; 11 Global Health Research Center. Guangdong Provincial People’s Hospital, Guangdong Academy of Medical Sciences, Guangzhou, China

**Keywords:** Epidemiology, Nutrition

## Abstract

**Background:**

Postmenopausal women tend to experience significant changes in body composition, particularly abdominal adipose tissue (AAT) deposition patterns, which are hypothesized to be critical factors influencing future chronic disease risk. The level of protein intake to maintain or achieve a more favorable body composition for health in postmenopausal women is a central, largely unanswered question relating to the appropriateness of current dietary guideline recommendations for sufficient protein intake (set at 0.8 g/kg/day).

**Objective:**

To estimate the hypothetical effect of a range of protein intake levels on 3-year mean changes in body composition measures in postmenopausal women.

**Methods:**

We analyzed data from 3789 postmenopausal women aged 50–79 enrolled in the Women’s Health Initiative (WHI) to emulate a 3-year target trial of adhering to increasing levels of protein intake: ≥0.8 g/kg/d, ≥1.0 g/kg/d, ≥1.2 g/kg/d, and ≥1.5 g/kg/d. All participants had repeated Dual X-Ray Absorptiometry (DXA) scans with derived abdominal visceral (VAT) and subcutaneous adipose tissue (SAT). The measured differences in average levels of VAT, SAT, and other body composition measures determined at end of follow-up were estimated with the parametric-g formula.

**Results:**

Over 3 years, hypothetical interventions of increasing levels of dietary protein intake are estimated to have dose-dependent reductions in abdominal VAT, SAT, and overall body fat, and increases in lean soft tissue, with potential benefits observed at ≥1.2 g/kg/day and the greatest estimated benefit at ≥1.5 g/kg/day of dietary protein. Compared to no intervention, if all participants hypothetically adhered to a total daily protein intake of ≥1.5 g/kg/day over 3 years, they would be estimated to have lower levels of VAT (−13.1 cm^2^, 95% Confidence Interval [CI] −18.9, −7.3), SAT (−25.3 cm^2^, 95% CI −39.7, −11.0), total body fat % (−1.0%, 95% CI −1.7, −0.3), body weight (−2.5 kg, 95% CI −3.7, −1.2) and greater lean soft tissue % (0.9%, 95% CI 0.3, 1.6) over 3 years.

**Conclusion:**

This hypothetical emulated intervention suggests that postmenopausal women who maintain a hypothetical total protein intake of at least 1.2 g/kg/day could experience beneficial changes in abdominal VAT, SAT, and overall body composition over three years, with even greater estimated benefits observed at an intake of 1.5 g/kg/day. These findings suggest that protein intake higher than guideline recommendations may better support healthier body composition and lower chronic disease risk in postmenopausal women.

## Introduction

During the menopausal transition and post-menopausal period, women commonly experience an age-related decline in skeletal muscle mass and an increase in adiposity, particularly around the abdomen [1–5]. This combination of higher adiposity, particularly abdominal visceral adipose tissue (VAT) or proxy measures, and decreased muscle mass comprise the evaluation of sarcopenic obesity and are individually linked with decreased physical functions and quality life, and increased risks of all-cause mortality and chronic cardiometabolic diseases [6–10]. As such, preventing accumulation of excess VAT and overall adiposity, and loss of skeletal muscle is recognized as a vital element in promoting healthy aging [11, 12].

Adequate protein consumption has been shown to slow sarcopenia onset, improve weight management, and increase cardiovascular function [13–16]. Observational studies have reported the relation between higher protein intake and the maintenance of optimal body composition in older adults [17–20], with some studies showing higher protein intake is associated with reduced lean muscle loss [21], a lower incidence of frailty [22], and improved physical function [23]. Most randomized controlled trials (RCTs) on this topic have been short-term and have not provided conclusive evidence [24]. In 2013, the PROT-AGE expert group recommended that older adults consume at least 1.0 to 1.2 g/kg/day of protein to optimize body composition and function [25], which is higher than the general dietary guidance for this population which is to consume at least 0.8 g/kg/day of protein [26, 27]. The potential impact of these recommended higher levels has not been documented.

To address this gap, we conducted a target trial emulation analysis. We estimated the hypothetical effects of multi-year protein intake interventions on body composition, including abdominal visceral adipose tissue (VAT), subcutaneous adipose tissue (SAT), total body fat, lean soft tissue mass, and body weight in postmenopausal women.

## Methods

### Target trial specification and treatment strategies

**Fig. 1:**
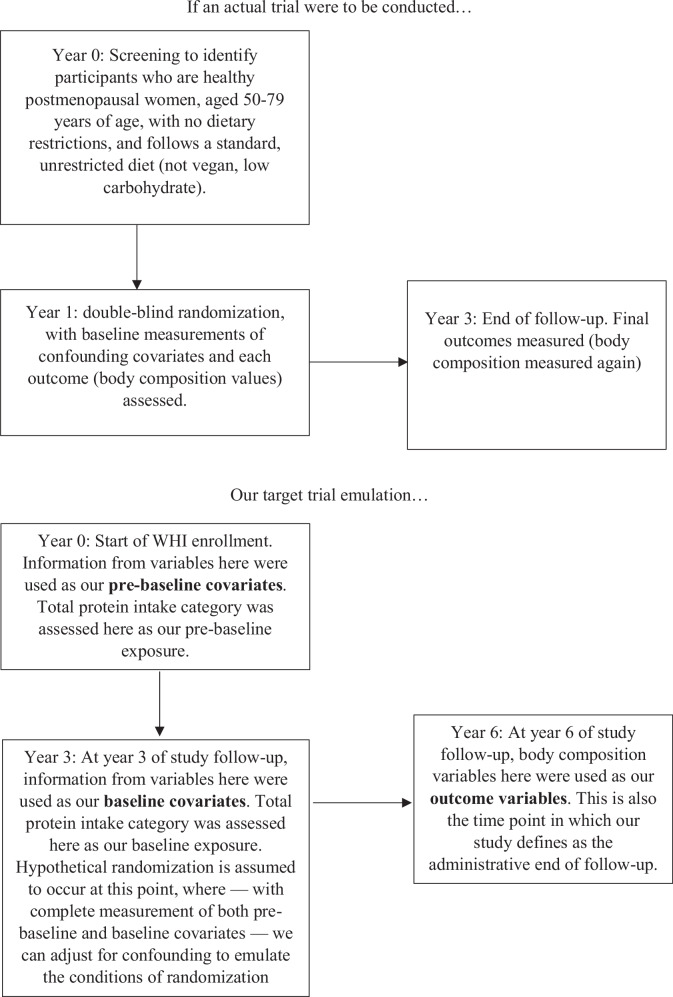
An illustration demonstration how pre-baseline covariates, baseline covariates, and outcomes were defined with respect to our study’s timeline.

**Fig. 2:**
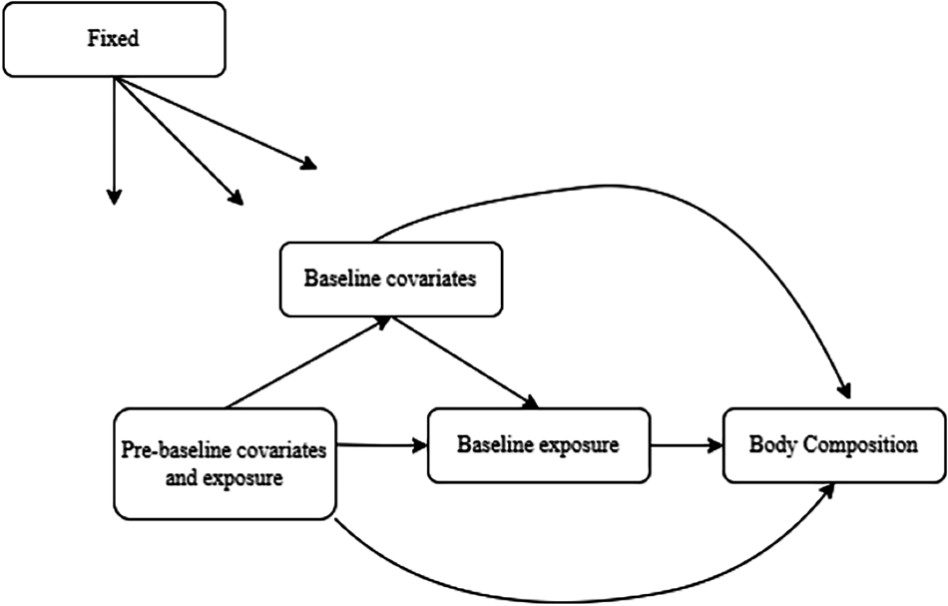
A causal directed acyclic graph depicting the assumed relation between total protein intake levels, pre-baseline, and baseline variables, and outcome. Baseline exposure: Baseline total protein intake (categorized). Pre-baseline covariate and exposure: Pre-Baseline confounding variables which include age, education, family income, race/ethnicity, coronary heart disease history, diabetes history, all-cause cancer history, alcohol intake, smoking status, HEI2020 dietary score, total daily energy intake, total protein intake, and sleeping hours. Baseline covariates: Baseline versions of all confounding variables from pre-baseline with exception of total protein intake variables. Body Composition: Body composition outcome variables at end of follow up, which include primary outcomes (VAT, SAT, total body fat percent, total lean soft tissue mass percent, and total bodyweight) and secondary outcomes (total arm fat/lean mass, total leg fat/lean mass, trunk fat/lean mass, android fat and gynoid fat mass).

We estimated the effect of hypothetical pragmatic interventions of increasing total protein intake on body composition using the emulated target trial framework. Using data collected prospectively every three years in the WHI, we established the baseline (time zero for the analysis) as the date of the initial 3-year visit. This leveraged an additional cycle of data before the analytic baseline, referred to as pre-baseline (i.e., data collected at enrollment at year 0), which enhances the emulation of randomization by applying two rounds of confounding adjustment via standardization. Assuming protein intake reported from the Food Frequency Questionnaire (FFQ) reflected the participants’ usual actual intake over the three years [28], utilizing a pre-baseline adjustment for year 0 covariates additionally helps minimize any potential differences between our study participants in the analytic process. A directed-acylic graph with selection rationale is presented in Figs. 1 and 2.

Protein intake interventions were defined to match protein intake ranges established in the PROT-AGE study [25]. These intake levels were [1]: ≥0.8 g/kg/d [2], ≥1.0 g/kg/d [3], ≥1.2 g/kg/d, and [4] ≥1.5 g/kg/d. The control group (i.e., the natural course) was defined as the protein intake that occurred throughout the study duration of three years without any active and specific intervention and includes women of all protein intake levels (Details of our intervention strategies are outlined in Table 1 protocol).

**Table 1:** Emulation of target trial of total protein intake interventions using observational data from the 1993–1998 Women’s Health Initiative Observational Study’s DXA sub-cohort.

	Target Trial Specification	Target Trial Emulation
**Aim**	To estimate the effect of different thresholds of total protein intake (g/kg of body weight/day) on changes in body composition of post-menopausal women (adiposity and lean muscle mass) over three years.	Same.
**Eligibility Criteria**	Postmenopausal women aged 50-79, with no dietary restrictions (no strict vegan or vegetarians), and no history of major chronic co-morbid conditions such as diabetes or cancer.	Same. Additionally, eligible individuals were also required to have complete data on total protein intake from the WHI food frequency questionnaire at both pre-baseline and baseline years.
**Intervention Strategies**	Participants are randomly assigned to initiate the 5 following strategies of total daily protein intake levels at baseline and adhere through the follow up period: 1. No intervention, which is defined as usual total protein intake, also referred to as natural course. 2. ≥0.8 g/kg/day 3. ≥1.0 g/kg/day 4. ≥1.2 g/kg/day 5. ≥1.5 g/kg/day	Same.
**Intervention Assignment**	Individuals are randomly assigned to a protein intake category intervention at baseline.	Random assignment emulation is achieved by adjusting/standardizing for pre-baseline and baseline covariates to create approximate exchangeability at baseline
**Outcome**	Primary Outcome: Body composition parameters evaluated at the end of the follow-up period include visceral adipose tissue (VAT), abdominal subcutaneous adipose tissue (SAT), weight, body fat percentage, and percentage of lean muscle mass mass. Secondary Outcomes: Body composition measures assessed at the end of the follow-up include trunk fat, trunk lean mass, android fat mass, gynoid fat mass, and mass of the appendicular regions (total arm fat mass, total leg fat mass, total arm lean mass, and total leg lean mass).	Same as specified.
**Follow-Up**	From randomization until the outcome measurement at 3 years or until the first occurrence of death, loss to follow-up, or the administrative end of follow-up.	Same as specified.
**Causal Contrast**	Both intent-to-treat and per-protocol effect.	Observational equivalent of the per-protocol effect.
**Statistical Analysis**	A per-protocol analysis would involve employing the g-formula to compare average body composition measures at the end of follow-up among groups receiving each intervention strategy. This comparison would be adjusted for baseline variables typically associated with each outlined strategy, as well as any potential loss to follow-up incurred.	Equivalent to per-protocol analysis.

### Study population

**Fig. 3:**
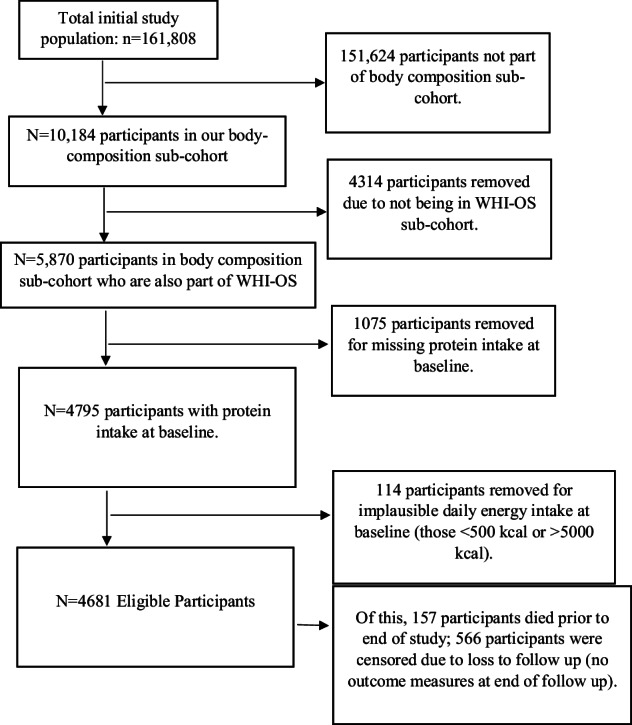
Selection of eligible participants for the target trial emulation of total protein intake and body composition among postmenopausal women from the Women’s Health Initiative (WHI).

Between 1993 and 1998, postmenopausal women aged 50–79 years were enrolled in the WHI clinical trials (CT) and observational study (OS) across 40 clinical centers in the U.S., totaling 161,808 participants [29]. Before commencement, all procedures and protocols underwent approval by the institutional review boards at each participating institution. Written, informed consent was obtained from all participants. Upon enrollment, the WHI administered comprehensive questionnaires on personal and family medical history, socio-demographics, and lifestyle characteristics, conducted in-person clinical examinations, and collected biological specimens [30]. Each year following baseline, participants completed annual questionnaires to update health information and identify new disease cases. A subset of participants from three WHI clinical centers (Pittsburgh, PA (*n* = 3590); Birmingham, AL (*n* = 3665); and Tucson/Phoenix, AZ (*n* = 3765)) were invited to join a sub-cohort study. This study involved body composition measurements via dual energy X-ray absorptiometry (DXA) at baseline and years 3, 6, and 9. The selection of these three DXA sites aimed to maximize the racial and ethnic diversity of the sub-cohort [31]. Out of 10,184 women who underwent DXA scans, 43% were from clinical trials and 57% from the observational study, all possessing relevant outcome variables. Due to the potential impact on protein intake and body weight caused by other interventions, clinical trial participants were excluded. Because participants with scans at year 9 represent a highly selected group of women who enrolled earliest in the WHI, these data are not representative of the full DXA sub-cohort. Therefore, our analyses include scans from years 0, 3, and 6 only. Pre-baseline variables are derived from year 0, baseline from year 3, and outcome variables of body composition from year 6, marking the end of follow-up of this hypothetical trial. Of the 5870 individuals in the observational study, 114 were excluded due to implausible daily energy intake at baseline (reporting below 500 kcals/2092 kJ or above 5000 kcals/20,920 kJ per day), resulting in 4681 participants eligible at baseline. Additionally, 157 participants died before or at the end of follow-up, and 566 were lost to follow-up by year 3, and were thus treated as censored. The final analytic cohort, therefore, of a total of 4681 post-menopausal women (Fig. 3).

### Protein intake measurement

Each woman in the WHI-OS study completed an FFQ at the beginning of the study and was reassessed at year 3. This FFQ comprised 122 items covering individual foods and food groups, along with 19 adjustment items and summary questions [32]. Daily nutrient intake was then calculated with the University of Minnesota’s Nutrient Data System for Research [33]. The energy-adjusted correlation coefficients between the FFQ and food records ranged from 0.2 to 0.7 for the estimated intakes of 30 nutrients, and 0.4 for protein [32]. Dietary protein intake was quantified in grams per day, which was subsequently divided by each participant’s weight in kilograms (kg) at the respective visit to calculate the protein intake per kilogram of body weight per day.

### Anthropometric body composition measurement

VAT, SAT, total body fat percentage, total lean soft tissue mass percentage, and total body weight were our primary outcomes of interest. Secondary outcomes included measures of trunk fat mass, trunk lean mass, appendicular regional mass, android fat mass, and gynoid fat mass. Whole-body DXA scans (QDR2000 and 4500 W; Hologic Inc., Marlborough, MA) were used to assess regional and total body composition. These scans followed standardized procedures and provided measurements of bone mineral density, lean soft tissue mass (kg), and fat mass (kg). Additionally, Hologic APEX 4.0 software was used to analyze archived DXA images from the WHI DXA sub-cohort, enabling estimation of abdominal VAT and SAT as well as other regional SAT depots. Specific measurement and validation procedures for VAT, SAT, and other anthropometric body composition measurements (such as weight and BMI) are detailed in our body composition measurements supplemental file (also see Chen et al and Bea et al.) [34, 35].

### Covariates

To account for confounding, we included the following measures: Age refers to the age at screening (calculated from participant’s birthdate). Education is the level of schooling attained, ranging from grade school (1–4 years) or (5–8 years), some high school (9–11 years), high school diploma or General Education Development (GED), vocational or training school, some college or associate degree, college graduate or baccalaureate degree, some post-grad or professional, master’s degree, to doctoral degree. Total family household income is segmented into brackets: less than $10k, $10k–$19,999k, $20k–$34,999k, $35k–$49,999k, $50k–$74,999k, $75k–$99,999k, $100k–$149,999k, and $150k or more. Race is determined via self-reported questionnaire, categorized according to NIH guidelines, including “American Indian/Alaska Native”, “Asian”, “Native Hawaiian/Other Pacific Islander (PI)”, “Black”, “White”, and “More than one race”. Ethnicity follows NIH guidelines as well, with options like “Not Hispanic/Latino”, “Hispanic/Latino”, and “Unknown/Not reported”. Marital status includes classifications such as never married, divorced/separated, widowed, presently married, and in a marriage-like relationship. Self-reported baseline cancer status was determined by whether participants had ever been diagnosed with cancer (excluding melanomas), while self-reported baseline diabetes status was based on whether participants had ever been diagnosed with diabetes. Alcohol consumption was defined by the number of servings per week of beer, wine, and/or liquor, based on medium serving sizes with standardized alcohol content measured in grams. Smoking habits were categorized as never, former, or current cigarette smokers. Diet quality and energy intake were assessed using a validated FFQ, which provided nutrient intakes that can be calculated for measures of total daily energy intake and Healthy Eating Index (HEI-2020) scores [36]. Sleep duration was characterized as the number of hours slept on an average night during the previous four weeks. This assessment encompassed various aspects of physical activity, including strength for lifting, carrying, stooping, bending, and climbing stairs, as well as the ability to walk without difficulty and perform self-care tasks [37]. Physical activity duration was assessed through a questionnaire detailing the duration and intensity of recreational activities or exercises (mild, moderate, and vigorous). This information enabled the calculation of metabolic equivalent (MET) levels for each activity, resulting in MET-hours per week. These values were then combined to determine the duration and intensity of physical activity via the MET-hours per week [38]. Depressive symptoms were evaluated using the Center for Epidemiological Studies (CES-D) depression scale, which ranges from 0 to 1, with higher scores indicating a greater likelihood of experiencing depressive symptoms. All covariates were defined and assessed at both pre-baseline and baseline.

### Parametric g-formula

The parametric g-formula (James Robins, 1986) was used to estimate the marginal causal effects analogous to those derived from randomized controlled trials [39]. If the identifiability conditions such as exchangeability, positivity, consistency, and absence of model misspecification, the parametric g-formula method provides an unbiased estimate for the average standardized outcome if the specific intervention of interest (i.e. specific levels of protein intake) were administered to every individual in the study [40]. The method involves the following steps: First, the distributions of the exposure levels (protein intake), confounders, outcome measures (body composition), and the censoring reasons are estimated using parametric models (e.g., linear or logistic regression) with history of the exposures and confounders and baseline values of confounders included. Second, confounder data are simulated based on a random sample of participants and the parametric confounder distributions. This estimates the conditional means of the outcomes according to confounder distributions and allows the setting of dietary protein intakes to the intervention strategy using Monte Carlo simulation to approximate the weighted average mean outcome measure. The simulations are conducted for each dietary intervention separately using the same parametric models. The standardized means for each intervention are estimated and used to estimate absolute measures of effect. We utilized bootstrap non-parametric sampling with 500 resamples to derive 95% CI for our main and sensitivity analyses. Comparisons were made between estimated treatment outcomes (end-of-follow-up body composition values) and expected outcomes under the natural course (no intervention). Our emulation adhered to the same eligibility criteria and intervention assignments for protein intake as outlined in Table 1 of our target trial protocol. Code availability: The data that supports the findings of this study will be made available upon reasonable request. All analyses were conducted using SAS (version 9.4, SAS Institute, Inc., Cary, NC).

### Ethics approval and consent to participate

This research was conducted using de-identified secondary data obtained from the WHI following approval of a data access request. All participants in the WHI provided informed consent at enrollment. Because this study involved secondary analysis of existing, de-identified data, no new participant contact had occurred and therefore additional institutional review board approval was not required.

## Results

Table 2 presents the pre-baseline characteristics of our post-menopausal women in the final analytical sample. On average, participants were 63.4 years old, predominantly white (83.0%), and non-Hispanic/Latino (7.4% Hispanic/Latino), with more than a third having vocational training or some college/associate degree (35.8%). The majority had a total family income of less than $35,000 annually (50.2%), were presently married (61.8%), and predominantly non-smokers (15.6% were smokers).

**Table 2:** Pre-baseline characteristics of *n* = 4681 eligible participants in the 1993–1998 WHI DXA-sub cohort for hypothetical total protein intake interventions.

Age, years^a^	63.4 (7.4)
Race categories for NIH reporting^b^	
American Indian/Alaska Native	48 (1.0)
Asian	22 (0.5)
Black	510 (10.6)
White	3982 (83.0)
More than one race	29 (0.6)
Ethnicity categories for NIH reporting^b^	
Hispanic/Latino	350 (7.4)
Total Family Household Income^b^	
Less than $35,000	2325 (50.2)
$35,000 to <$75,000	1544 (33.3)
$75,000 or more	557 (12.0)
Highest Educational Attainment^b^	
High school diploma or GED or less	1441 (30.3)
Vocational or some college/Associate’s	1705 (35.8)
College graduate to professional	873 (18.8)
Master’s degree or above	739 (15.5)
Marital Status^b^	
Never married	188 (3.9)
Divorced or separated	659 (13.8)
Widowed	926 (19.4)
Presently married	2949 (61.8)
Marriage-like relationship	50 (1.1)
Total daily protein intake category^b,^^c^	
0.5–0.8 g/kg/day	1967 (41.0)
>0.8–1.0 g/kg/day	922 (19.2)
>1.0–1.2 g/kg/day	750 (15.6)
>1.2–1.5 g/kg/day	631 (13.2)
>1.5 g/kg/day	525 (11.0)
Sleeping Duration^b^	
5 or less hours	430 (9.0)
6 h	1272 (26.7)
7 h	1807 (38.0)
8 h	1028 (21.6)
9+h	222 (4.7)
Dietary intake score, HEI-2015^a^	65.8 (10.6)
Dietary energy in kcal^a^	1575.7 (729.0)
Dietary energy in kJ^a^	6592.7 (3050.1)
Alcohol servings per week^a^	1.9 (4.4)
Smoking Habit^b^	
Yes	336 (15.6)
Diabetes status^b^	
Yes	296 (6.2)
Cancer status^b^	
Yes	450 (9.5)
Weight, kg^a,d^	70.1 (15.0)
SAT, cm^2a,d^	358.5 (135.2)
VAT, cm^2a,d^	153.6 (81.7)
Bodyfat, %^a,d^	54.2 (7.2)
Lean soft tissue mass, %^a,d^	42.8 (7.5)

*WHI* Women’s Health Initiative, *NIH* National Institutes of Health, *GED* General Education Diploma, *HEI* Healthy Eating Index, *DXA* Dexa Sub-cohort.

^a^Mean (SD).

^b^*n* (%).

^c^Categorized from a computed total daily protein intake divided by the participant’s weight in kilograms (kg).

^d^Mean outcome measure at baseline.

Overall, participants consumed an average of 1.9 servings of alcohol per week and averaged a dietary intake quality score (HEI 2020 index) of 65.8. Most women (41.0%) reported consuming between 0.5 and 0.8 g/kg/day of total protein, while 19.2% consumed more than 0.8 to 1.0 g/kg/day. Additionally, 15.6% consumed more than 1.0 to 1.2 g/kg/day, 13.2% consumed more than 1.2 to 1.5 g/kg/day, and 11.0% consumed more than 1.5 g/kg/day. On average, participants slept for 7 hours. Less than 10% of participants had diabetes or cancer at pre-baseline (6.2% diagnosed with diabetes and 9.5% diagnosed with cancer). The average weight was 70.1 kg, with an average SAT of 358.5 cm^2^, VAT of 153.6 cm^2^, body fat percentage of 54.2%, and lean soft tissue mass percentage of 42.8%.

Table 3 presents the primary findings regarding total body weight, visceral adipose tissue (VAT), subcutaneous adipose tissue (SAT), total body fat percentage (%), and total lean soft tissue mass percentage (%) according to levels of the hypothetical interventions. These estimates reflect the expected levels if all participants in the study were to follow the designated total protein intake interventions outlined in Table 1 of the emulation strategy.

**Table 3:** Estimated levels of Visceral Adipose Tissue (VAT), Subcutaneous Adipose Tissue (SAT), total body fat percentage, total lean Soft Tissue mass percentage, and total bodyweight at end of follow-up after 3 years of hypothetical protein intake interventions in post-menopausal women in the 1993–1998 Women’s Health Initiative (WHI) observational study DXA sub-cohort.

Interventions		VAT (cm^2^)	SAT (cm^2^)	Total Body Fat (%)	Total Lean Soft Tissue mass (%)	Weight (kg)
0	Natural Course (reference, no intervention)	169.6 (165.5, 173.7)	367.5 (359.8, 375.2)	43.2 (42.8, 43.6)	53.9 (53.5, 54.3)	71.4 (70.5, 72.2)
1	≥0.8 g/kg/day	174.4 (171.7, 177.2)	372.8 (365.2, 380.4)	43.5 (43.1, 43.9)	53.6 (53.2, 53.9)	72.1 (71.1, 73.0)
2	≥1.0 g/kg/day	169.3 (166.2, 172.5)	364.1 (355.8, 372.3)	43.1 (42.7, 43.5)	53.9 (53.5, 54.3)	71.2 (70.2, 72.1)
3	≥1.2 g/kg/day	164.2 (159.8, 168.6)	355.3 (344.5, 366.1)	42.8 (42.3, 43.2)	54.3 (53.8, 54.7)	70.3 (69.2, 71.3)
4	≥1.5 g/kg/day	156.5 (149.7, 163.4)	342.2 (326.1, 358.3)	42.2 (41.5, 42.9)	54.8 (54.2, 55.5)	68.9 (67.5, 70.3)
**Average Treatment Effects**						
Intervention 1 vs 0		4.8 (2.4, 7.3)	5.3 (1.4, 9.1)	0.4 (0.2, 0.5)	−0.3 (−0.5, −0.2)	0.7 (0.2, 1.2)
Intervention 2 vs 0		−0.3 (−2.5, 2.0)	−3.5 (−8.1, 1.2)	0.0 (−0.3, 0.3)	0.0 (−0.2, 0.2)	−0.2 (−0.7, 0.3)
Intervention 3 vs 0		−5.4 (−8.8, −2.1)	−12.2 (−20.4, −4.0)	−0.4 (−0.8, 0.0)	0.4 (0.0, 0.7)	−1.1 (−1.9, −0.3)
Intervention 4 vs 0		−13.1 (−18.9, −7.3)	−25.3 (−39.7, −11.0)	−1.0 (−1.7, −0.3)	0.9 (0.3, 1.6)	−2.5 (−3.7, −1.2)

Fully adjusted outcome model includes pre-baseline and baseline confounders: age, education, race, ethnicity, income, alcohol intake, sleeping duration, smoking status, CES-D depression score, HEI 2015 diet score, total physical activity intensity (MET-hrs), marital status, diabetes at baseline, and cancer at baseline. Additionally, pre-baseline intake of daily total protein and both pre-baseline and baseline versions of our outcome measurements were also adjusted.

With each step of higher daily protein intake intervention there were lower estimates of VAT, SAT, total body fat percentage, and total weight and higher total lean soft tissue percentage at the end of the follow-up, with benefits observed at ≥1.2 g/kg/day and the greatest estimated benefit at ≥1.5 g/kg/day of dietary protein intake. Specifically, compared to the natural course, if all post-menopausal women in the analytic sample were to adhere to a total daily protein intake of ≥1.2 g/kg/day over 3 years, they would be estimated to have lower levels of VAT (−5.4 cm^2^, 95% CI −8.8, −2.1), SAT (−12.2 cm^2^, 95% CI −20.4, −4.0), total body fat % (−0.4%, 95% CI −0.8, 0.0), body weight (−1.1 kg, 95% CI −1.9, −0.3) and higher lean soft tissue mass % (0.4%, 95% CI 0.0, 0.7) over 3 years. Similarly, at the highest level at ≥1.5 g/kg/day over 3 years, they would have lower levels of VAT (−13.1 cm^2^, 95% CI −18.9, −7.3), SAT (−25.3 cm^2^, 95% CI −39.7, −11.0), total body fat % (−1.0%, 95% CI −1.7, −0.3), body weight (−2.5 kg, 95% CI −3.7, −1.2) and higher lean soft tissue mass % (0.9%, 95% CI 0.3, 1.6) over 3 years.

The estimated effects of all five interventions on regional measures of adiposity and lean soft tissue mass, including the trunk, appendicular, android, and gynoid regions aligned with that of main outcomes (VAT, SAT, body fat percentage, lean soft tissue mass percentage, and weight), where a higher intake of total daily protein provides protective benefits over 3 years [See supplementary file (S1), Tables 1–4]. However, when examining the regional measures of lean soft tissue, it is clear the results of the overall higher percentage of total lean soft tissue are due to the lower level of adipose tissue, rather than higher absolute levels of lean soft issue. Supplementary Tables 5–8 (S1) present sensitivity analyses using categorical protein intake levels, showing qualitatively similar patterns to our main threshold intervention results. Table 9 (S1) presents categorical levels of protein intake, further stratified by each group’s mean total daily protein intake in grams, total daily energy intake in kcals and kJ, and the mean percentage of total energy intake derived from protein intake. Table 10 provides a cross-tabulation stratifying participants by commonly used BMI categories and average daily protein intake. Tables 11–16 report average energy intake by baseline quintiles of body composition (VAT, SAT, bodyweight, body fat %, lean mass %) and age group (<65, ≥65). Supplemental File S2 presents all of our outcomes with an additional adjustment for total daily energy intake. Supplemental File S3 includes visualizations of our main outcomes by the natural course and four separate hypothetical intervention strategies.

## Discussion

We conducted a target trial emulation to estimate the effect of hypothetical dietary interventions of higher total protein intake levels (g/kg/day) over 3 years on changes in body composition in 4681 postmenopausal women. The results suggest that initiating a diet with higher levels of protein intake among postmenopausal women would have a dose-response-like outcome on body composition with lower levels of VAT, SAT, total body fat, and higher lean mass % with estimated benefits at ≥1.2 g/kg/day relative to no intervention.

To date, there are a few large-scale prospective cohort studies that have investigated the relation between protein intake and body composition in older adults. Houston et al. found that older adults aged 70–79 in the Health, Aging, and Body Composition Study who were in the highest quintile of protein intake (1.2 ± 0.4 g/kg/day) lost 40% less lean mass over three years compared to those in the lowest quintile (0.8 ± 0.3 g/kg/day) over 3 years of follow-up [21]. Meng et al. studied a cohort of 862 community-dwelling women with an average age of 75 over five years and found positive correlations between baseline protein intake and total body weight, appendicular bone-free lean mass, and upper arm muscle area [41]. Women in the lowest quintile of protein intake (1.22 ± 0.45 g/kg/day) had the lowest levels of total body weight, appendicular bone-free lean mass, and upper arm muscle area, while those in the highest quintile of protein intake (1.64 ± 0.44 g/kg/day) had the highest levels in these measures. Similarly, Martinez et al. found that the highest quintile of protein consumption (1.22–2.02 g/kg; average 1.35 g/kg) was associated with the highest levels of lean mass in postmenopausal women of the WHI when compared to the lowest quintile consumers (0.54–0.89 g/kg; average 0.79 g/kg), though the analysis was cross-sectional despite using prospective cohort data [42].

Studies about the exact values of protein intake recommendations are limited. Some non-interventional studies have recommended raising the daily protein intake limit to over 0.8 g/kg/day without specifying exact values [17, 43]. Others recommended ranges of 1.0–1.5 g/kg/day to better optimize health [15, 44–46]. There have been smaller interventional studies examining the effects of varying protein intake per kilogram on older adults’ body composition, but most have focused on participants with overweight or obesity. To examine the effects of higher protein intake on body composition during weight loss, Backx et al. randomized 61 adults with overweight or obesity (average age 63) to hypocaloric diets with protein intakes at either 0.9 g/kg/day (placebo group) or 1.7 g/kg/day (high protein group) but did not find large significant differences between the two groups [47]. Over 12 weeks with a 25% energy intake restriction, the placebo group experienced a lean mass decline of 2.1 ± 1.4 kg, while the high protein group saw a decline of 1.8 ± 2.2 kg. Sammarco et al. conducted a small interventional study of dietary protein enrichment on body composition during weight loss in middle-aged sarcopenic women with obesity (ages 41–74) [48], showing significant improvement in lean body mass preservation in those randomized to a hypocaloric high-protein diet of 1.2–1.4 g/kg/day vs those in the placebo hypocaloric group (0.8–1.0 g/kg/day) after 4 months. Similarly, Wright et al. also conducted a 12-week parallel-design, interventional study which found that a 12-week eucaloric diet supplemented with whole eggs (1.4 g/kg/day total protein intake) attenuated lean body mass loss and resulted in modest overall weight loss in older adults (average age 70) with overweight or obesity vs a normal protein diet of 0.8 g/kg/day of total intake [49].

Our study’s findings align with these recommendations, suggesting that higher protein intake (≥1.2 g/kg/day) may improve body composition in postmenopausal women over 3 years. This is especially important given the relation between body composition and chronic diseases among older individuals [50]. Overall, research has consistently underscored the importance of factors like proper nutrition, hydration, and micronutrient intake in optimizing nutritional status, particularly among older individuals at risk of adverse body composition-related outcomes such as sarcopenia [51–54]. Our study suggests that increasing protein intake above currently recommended levels (0.8 g/kg/day) is another factor that would improve body composition. In our sensitivity analyses, we additionally explored adjusting for total daily energy intake (Supplemental Table S2), which showed slightly attenuated estimated effects of each protein intake intervention. This may suggest that total energy intake also plays a role in influencing both adiposity gain and lean tissue loss. For instance, in the case of visceral adipose tissue (VAT), our primary results (Table 3) estimated a difference of −5.4 cm², while the energy-adjusted model showed a reduced estimate of −4.4 cm². The decision to adjust for daily energy intake was driven by its potential role as a confounder. As shown in Table 9 of Supplementary File S1, the proportion of energy from protein increased modestly across intake groups—ranging from 16.2% in those consuming ≤0.8 g/kg/day to 19.0% in those consuming >1.5 g/kg/day—suggesting little reason for confounding, which is also reflected in our energy-adjusted analyses where changes in our primary estimates were relatively minor. We explored confounding by body composition: Table 10 examined protein intake by BMI, and Tables 11–16 assessed energy intake across quintiles of VAT, SAT, body weight, body fat, and lean mass.

Strengths of this study include the large, well-defined and characterized population of the WHI and the detailed, valid, body composition measurements repeated over time, which provides the ideal structure to leverage observational data to estimate causal effects over a longer period than any interventional study would reasonably be able to do. The application of the g-formula approach provides population-level effect estimation, contrasting with conventional adjustment-based analyses [39]. This approach offers insights for decision-making and guidelines, illustrating the potential impact of different levels of protein intake on changes in body composition in postmenopausal women.

However, the target trial approach does not eliminate the possibility of unmeasured confounding, model misspecification, and inaccurate measurement of variables. These assumptions are challenging to verify in observational study, potentially leading to biased estimates if violated. For example, violations of stable unit treatment value assumption, such as interference between individuals or differing treatment experiences, can lead to positively biased estimates and threaten the validity of our causal interpretation [55]. Potential model misspecification is always a broad concern, though our natural course estimates track closely with observed outcomes, suggesting no gross model misspecification under no-treatment. Since our g-formula estimates are theoretical analogs to per-protocol effect estimation, it’s important to note that per-protocol effect estimates in randomized trials are also susceptible to measurement error, as adherence to the assigned intervention is often assessed through questionnaire responses.

Moreover, while our study estimates the effects of specific protein intake thresholds, these levels may not be realistic or economically feasible for all individuals, as personal tolerances, dietary habits, and the typically higher cost of protein-rich foods could make it unlikely for some participants to consume the hypothetically assigned amounts. These thresholds were based on actual body weight rather than ideal body weight, as is sometimes recommended in clinical practice for individuals with obesity. Although we observed hypothetical effects like 2.5 kg weight loss, 1% lower body fat, and 1% higher lean mass, these may not be clinically meaningful. We also did not distinguish between animal and plant protein sources, given the current lack of consensus on their influences on body composition in older adults. Errors associated with FFQs and other recall-based methods, such as the fixed list of foods, mean that not all sources of animal and plant-based protein may have been thoroughly identified and assessed in the WHI FFQ. This limitation could potentially raise concerns about the generalizability of the results if stratified by protein type [28, 32]. Additionally, we also do not have a validated biomarker-based measure of protein intake, such as urinary nitrogen; therefore, we additionally relied on the assumption that the self-reported FFQ would provide a reasonably reliable estimate of our exposure. Lastly, the results of our study primarily pertain to postmenopausal women, who comprise our cohort, may not be fully representative of the broader U.S. population.

## Conclusion

We estimated the hypothetical effects of different levels of daily protein intake on body composition measures relevant to healthy aging and chronic disease risk in postmenopausal women. Our results estimate that a hypothetical protein intake of at least ≥1.2 g/kg/day could lead to lower levels of VAT, regional SAT, overall body fat percentage, and body weight, along with higher relative lean mass over three years, with even greater potential benefits observed at an intake of 1.5 g/kg/day. These findings suggest that higher protein intake may promote healthier body composition in postmenopausal women.

## Supplementary information

Supplemental Files Combined PDF

Supplementary information

## Data Availability

The datasets generated during and/or analyzed during the current study are available from the corresponding author on reasonable request.
