# Supplementary material for: Estimating the effect of hypothetical dietary protein interventions on changes in body composition of postmenopausal women over 3 years using data from the Women’s Health Initiative (WHI) Study: an emulated target trial
Source: Int J Obes (Lond). 2026 Jan 9;50(3):609–17. doi: 10.1038/s41366-025-01978-0 (PMC12965867; doi:10.1038/s41366-025-01978-0)
Supplement: Supplementary file 1 — Supplemental Files Combined PDF [file 41366_2025_1978_MOESM1_ESM.pdf]

Supplemental File S1

S1 Table 1: Estimated levels of total body fat and lean mass at end of follow-up after 3 years of hypothetical protein intake interventions in post-menopausal women in the 1993 – 1998 Women's Health Initiative (WHI) observational study DXA sub-cohort.

Interventions		Total body fat mass (kg)	Total lean soft tissue mass (kg)
0	Natural Course (reference, no intervention)	31.7 (31.3, 32.2)	37.5 (37.2, 37.8)
1	≥ 0.8 g/kg/day	32.4 (31.8, 33.0)	37.7 (37.4, 37.9)
2	≥ 1.0 g/kg/day	31.6 (31.1, 32.2)	37.4 (37.2, 37.6)
3	≥ 1.2 g/kg/day	30.9 (30.1, 31.7)	37.1 (36.8, 37.4)
4	≥ 1.5 g/kg/day	29.7 (28.4, 31.0)	36.7 (36.3, 37.1)
**Average Treatment Effects**			
Intervention 1 vs 0		0.7 (0.3, 1.0)	0.2 (0.0, 0.3)
Intervention 2 vs 0		-0.1 (-0.5, 0.3)	-0.1 (-0.3, 0.0)
Intervention 3 vs 0		-0.9 (-1.6, -0.2)	-0.4 (-0.7, -0.1)
Intervention 4 vs 0		-2.0 (-3.3, -0.8)	-0.8 (-1.2, -0.4)

Fully adjusted outcome model includes pre-baseline and baseline confounders: age, education, race, ethnicity, income, alcohol intake, sleeping duration, smoking status, CES-D depression score, HEI 2015 diet score, total physical activity intensity (MET-hrs), marital status, diabetes at baseline, and cancer at baseline. Additionally, pre-baseline intake of daily total protein and both pre-baseline and baseline versions of our outcome measurements were also adjusted.

S1 Table 2: Estimated levels of trunk adipose tissue and lean Soft Tissue mass at end of follow-up after 3 years of hypothetical protein intake interventions in post-menopausal women in the 1993 – 1998 Women's Health Initiative (WHI) observational study DXA sub-cohort.

Interventions		Trunk Adipose Tissue (kg)	Trunk Lean Soft Tissue mass (kg)
0	Natural Course (reference, no intervention)	14.7 (14.4, 15.1)	19.7 (19.5, 19.9)
1	≥ 0.8 g/kg/day	15.1 (14.8, 15.4)	19.8 (19.6, 20.0)
2	≥ 1.0 g/kg/day	14.7 (14.4, 15.0)	19.6 (19.5, 19.8)
3	≥ 1.2 g/kg/day	14.3 (13.9, 14.7)	19.5 (19.3, 19.7)
4	≥ 1.5 g/kg/day	13.6 (13.0, 14.3)	19.3 (19.1, 19.5)
**Average Treatment Effects**			
Intervention 1 vs 0		0.3 (0.1, 0.6)	0.1 (0.0, 0.2)
Intervention 2 vs 0		-0.1 (-0.3, 0.2)	0.0 (-0.1, 0.0)
Intervention 3 vs 0		-0.5 (-0.8, -0.1)	-0.2 (-0.2, -0.1)
Intervention 4 vs 0		-1.1 (-1.7, -0.5)	-0.4 (-0.5, -0.3)

Fully adjusted outcome model includes pre-baseline and baseline confounders: age, education, race, ethnicity, income, alcohol intake, sleeping duration, smoking status, CES-D depression score, HEI 2015 diet score, total physical activity intensity (MET-hrs), marital status, diabetes at baseline, and cancer at baseline. Additionally, pre-baseline intake of daily total protein and both pre-baseline and baseline versions of our outcome measurements were also adjusted.

**S1 Table 3: Estimated levels of appendicular regional outcomes at end of follow-up after 3 years of hypothetical protein intake interventions in post-menopausal women in the 1993 – 1998 Women's Health Initiative (WHI) observational study DXA sub-cohort.**					
**Interventions**		**Total Leg Adipose Tissue (kg)**	**Total Leg Lean Soft Tissue mass (kg)**	**Total Arm Adipose Tissue (kg)**	**Total Arm Lean Soft Tissue mass (kg)**
0	Natural Course (reference)	11.7 (11.5, 12.0)	11.3 (11.2, 11.4)	4.5 (4.3, 4.6)	3.4 (3.4, 3.5)
1	≥ 0.8 g/kg/day	11.9 (11.6, 12.2)	11.4 (11.2, 11.5)	4.6 (4.4, 4.7)	3.5 (3.4, 3.5)
2	≥ 1.0 g/kg/day	11.8 (11.5, 12.0)	11.3 (11.1, 11.4)	4.4 (4.3, 4.5)	3.4 (3.3, 3.5)
3	≥ 1.2 g/kg/day	11.6 (11.3, 11.9)	11.2 (11.0, 11.3)	4.2 (4.0, 4.3)	3.4 (3.3, 3.4)
4	≥ 1.5 g/kg/day	11.4 (11.0, 11.8)	11.0 (10.9, 11.2)	3.8 (3.6, 4.1)	3.3 (3.2, 3.4)
**Average Treatment Effects**					
Intervention 1 vs 0		0.2 (0.0, 0.3)	0.0 (0.0, 0.1)	0.1 (0.0, 0.2)	0.0 (0.0, 0.1)
Intervention 2 vs 0		0.0 (-0.2, 0.2)	-0.1 (-0.1, 0.0)	-0.1 (-0.2, 0.0)	0.0 (0.0, 0.0)
Intervention 3 vs 0		-0.1 (-0.4, 0.1)	-0.2 (-0.2, -0.1)	-0.3 (-0.4, -0.1)	-0.1 (-0.1, 0.0)
Intervention 4 vs 0		-0.3 (-0.7, 0.1)	-0.3 (-0.5, -0.2)	-0.6 (-0.9, -0.3)	-0.2 (-0.3, -0.1)
Fully adjusted outcome model includes pre-baseline and baseline confounders: age, education, race, ethnicity, income, alcohol intake, sleeping duration, smoking status, CES-D depression score, HEI 2015 diet score, total physical activity intensity (MET-hrs), marital status, diabetes at baseline, and cancer at baseline. Additionally, pre-baseline intake of daily total protein and both pre-baseline and baseline versions of our outcome measurements were also adjusted.					

**S1 Table 4: Estimated levels of android and gynoid regional outcomes at end of follow-up after 3 years of hypothetical protein intake interventions in post-menopausal women in the 1993 – 1998 Women's Health Initiative (WHI) observational study DXA sub-cohort.**					
**Interventions**		**Android Adipose Tissue (kg)**	**Android Lean Soft Tissue mass (kg)**	**Gynoid Adipose Tissue (kg)**	**Gynoid Lean Soft Tissue mass (kg)**
0	Natural Course (reference)	2.5 (2.4, 2.6)	2.9 (2.9, 3.0)	5.5 (5.4, 5.6)	5.9 (5.8, 5.9)
1	≥ 0.8 g/kg/day	2.6 (2.5, 2.6)	3.0 (2.9, 3.0)	5.6 (5.5, 5.7)	5.9 (5.8, 6.0)
2	≥ 1.0 g/kg/day	2.5 (2.4, 2.6)	2.9 (2.9, 3.0)	5.5 (5.4, 5.6)	5.9 (5.8, 5.9)
3	≥ 1.2 g/kg/day	2.4 (2.3, 2.5)	2.9 (2.8, 2.9)	5.4 (5.3, 5.6)	5.8 (5.7, 5.9)
4	≥ 1.5 g/kg/day	2.3 (2.2, 2.4)	2.8 (2.7, 2.9)	5.3 (5.1, 5.5)	5.7 (5.7, 5.8)
**Average Treatment Effects**					
Intervention 1 vs 0		0.1 (0.0, 0.1)	0.0 (0.0, 0.1)	0.1 (0.0, 0.2)	0.0 (0.0, 0.1)
Intervention 2 vs 0		0.0 (-0.1, 0.0)	0.0 (0.0, 0.0)	0.0 (-0.1, 0.1)	0.0 (0.0, 0.0)
Intervention 3 vs 0		-0.1 (-0.2, 0.0)	0.0 (-0.1, 0.0)	-0.1 (-0.2, 0.0)	-0.1 (-0.1, 0.0)
Intervention 4 vs 0		-0.2 (-0.3, -0.1)	-0.1 (-0.1, 0.0)	-0.2 (-0.4, -0.1)	-0.1 (-0.2, -0.1)

Fully adjusted outcome model includes pre-baseline and baseline confounders: age, education, race, ethnicity, income, alcohol intake, sleeping duration, smoking status, CES-D depression score, HEI 2015 diet score, total physical activity intensity (MET-hrs), marital status, diabetes at baseline, and cancer at baseline. Additionally, pre-baseline intake of daily total protein and both pre-baseline and baseline versions of our outcome measurements were also adjusted.

S1 Table 5: Estimated levels of Visceral Adipose Tissue (VAT), Subcutaneous Adipose Tissue (SAT), total body fat percentage, total lean Soft Tissue mass percentage, and total bodyweight at end of follow-up after 3 years of hypothetical (categorical) protein intake interventions in post-menopausal women in the 1993 – 1998 Women's Health Initiative (WHI) observational study DXA sub-cohort.

Interventions		VAT (cm^2)	SAT (cm^2)	Total Body Fat (%)	Total Lean soft tissue mass (%)	Weight (kg)
0	Natural Course (no intervention)	170.0 (165.1, 174.9)	369.7 (362.1, 377.3)	43.3 (42.9, 43.7)	53.7 (53.4, 54.1)	71.5 (70.6, 72.3)
1	≤ 0.8g/kg/day (reference)	175.8 (170.3, 181.4)	380.1 (371.9, 388.3)	43.7 (43.3, 44.1)	53.4 (53.0, 53.8)	71.8 (71.0, 72.7)
2	> 0.8 but ≤ 1g/kg/day	170.3 (164.3, 176.3)	363.8 (355.4, 372.2)	43.3 (42.7, 43.9)	53.8 (53.2, 54.4)	71.6 (70.7, 72.5)
3	>1 but ≤ 1.2g/kg/day	165.7 (157.9, 173.4)	358.2 (346.5, 369.8)	43.1 (42.2, 44.0)	54.0 (53.1, 54.8)	71.2 (70.1, 72.2)
4	> 1.2 but ≤ 1.5g/kg/day	163.3 (154.6, 172.0)	356.1 (344.4, 367.7)	42.7 (41.8, 43.6)	54.4 (53.5, 55.2)	70.9 (69.9, 72.0)
5	> 1.5 g/kg/day	162.2 (150.9, 173.5)	345.7 (329.3, 362.2)	42.6 (41.5, 43.8)	54.4 (53.3, 55.5)	70.9 (69.5, 72.4)
**Average Treatment Effects**						
Intervention 2 vs 1		-5.5 (-11.9, 0.9)	-16.3 (-25.0, -7.5)	-0.4 (-1.1, 0.3)	0.4 (-0.2, 1.1)	-0.2 (-1.0, 0.5)
Intervention 3 vs 1		-10.2 (-18.9, -1.4)	-21.9 (-34.9, -9.0)	-0.6 (-1.6, 0.3)	0.6 (-0.3, 1.5)	-0.7 (-1.7, 0.3)
Intervention 4 vs 1		-12.6 (-22.8, -2.3)	-24.0 (-37.0, -10.9)	-1.0 (-2.0, 0.0)	0.9 (0.0, 1.9)	-0.8 (-1.9, 0.1)
Intervention 5 vs 1		-13.6 (-26.6, -0.6)	-34.3 (-53.0, -15.7)	-1.1 (-2.3, 0.2)	1.0 (-0.2, 2.2)	-0.9 (-2.3, 0.5)

Fully adjusted outcome model includes pre-baseline and baseline confounders: age, education, race, ethnicity, income, alcohol intake, sleeping duration, smoking status, CES-D depression score, HEI 2015 diet score, total physical activity intensity (MET-hrs), marital status, diabetes at baseline, and cancer at baseline. Additionally, pre-baseline intake of daily total protein and both pre-baseline and baseline versions of our outcome measurements were also adjusted.

S1 Table 6: Estimated levels of trunk adipose tissue and lean muscle mass at end of follow-up after 3 years of hypothetical (categorical) protein intake interventions in post-menopausal women in the 1993 – 1998 Women's Health Initiative (WHI) observational study DXA sub-cohort.

Assigned Protein Intake Interventions (Baseline)		Trunk Adipose Tissue (kg)	Trunk Lean Soft Tissue mass (kg)
0	Natural Course (no intervention)	14.8 (14.5,15.2)	19.7 (19.5,19.8)
1	≤ 0.8g/kg/day (reference)	15.3 (14.9,15.7)	19.8 (19.6,19.9)
2	> 0.8 but ≤ 1g/kg/day	14.9 (14.5,15.3)	19.8 (19.6,20.0)
3	>1 but ≤ 1.2g/kg/day	14.4 (13.8,14.9)	19.6 (19.4,19.8)
4	> 1.2 but ≤ 1.5g/kg/day	14.2 (13.6,14.7)	19.6 (19.4,19.8)
5	> 1.5 g/kg/day	13.9 (13.1,14.7)	19.2 (18.8,19.5)

Average Treatment Effects		
Intervention 2 vs 1	-0.3 (-0.8,0.0)	0.0 (-0.2,0.2)
Intervention 3 vs 1	-0.9 (-1.5,-0.3)	-0.1 (-0.4,0.1)
Intervention 4 vs 1	-1.1 (-1.7,-0.5)	-0.2 (-0.4,0.1)
Intervention 5 vs 1	-1.3 (-2.3,-0.4)	-0.6 (-1.0,-0.2)

Fully adjusted outcome model includes pre-baseline and baseline confounders: age, education, race, ethnicity, income, alcohol intake, sleeping duration, smoking status, CES-D depression score, HEI 2015 diet score, total physical activity intensity (MET-hrs), marital status, diabetes at baseline, and cancer at baseline. Additionally, pre-baseline intake of daily total protein and both pre-baseline and baseline versions of our outcome measurements were also adjusted.

S1 Table 7: Estimated levels of appendicular regional outcomes at end of follow-up after 3 years of hypothetical (categorical) protein intake interventions in post-menopausal women in the 1993 – 1998 Women's Health Initiative (WHI) observational study DXA sub-cohort.

Assigned Protein Intake Interventions (Baseline)		Total Leg Adipose Tissue (kg)	Total Leg Lean Soft Tissue mass (kg)	Total Arm Adipose Tissue (kg)	Total Arm Lean Soft Tissue mass (kg)
0	Natural Course (no intervention)	11.8 (11.6,12.0)	11.3 (11.2,11.4)	4.4 (4.3,4.6)	3.4 (3.4,3.5)
1	≤ 0.8g/kg/day (reference)	12.0 (11.8,12.3)	11.4 (11.2,11.5)	4.6 (4.4,4.8)	3.4 (3.4,3.5)
2	> 0.8 but ≤ 1g/kg/day	11.9 (11.7,12.2)	11.2 (11.1,11.4)	4.4 (4.2,4.5)	3.4 (3.4,3.5)
3	>1 but ≤ 1.2g/kg/day	11.6 (11.3,12.0)	11.3 (11.2,11.5)	4.4 (4.2,4.6)	3.4 (3.3,3.5)
4	> 1.2 but ≤ 1.5g/kg/day	11.5 (11.1,11.8)	11.2 (11.1,11.4)	4.2 (4.0,4.4)	3.4 (3.3,3.5)
5	> 1.5 g/kg/day	11.5 (11.0,11.9)	11.1 (10.9,11.3)	4.0 (3.7,4.3)	3.3 (3.2,3.4)
**Average Treatment Effects**					
Intervention 2 vs 1		-0.1 (-0.3,0.1)	-0.1 (-0.3,0.0)	-0.2 (-0.4,0.0)	0.0 (-0.1,0.0)
Intervention 3 vs 1		-0.3 (-0.7,0.0)	-0.1 (-0.2,0.1)	-0.2 (-0.4,0.0)	0.0 (-0.1,0.0)
Intervention 4 vs 1		-0.5 (-0.9,-0.2)	-0.1 (-0.3,0.1)	-0.4 (-0.6,-0.1)	0.0 (-0.1,0.0)
Intervention 5 vs 1		-0.5 (-1.1,0.0)	-0.3 (-0.5,0.0)	-0.6 (-0.9,-0.2)	-0.1 (-0.2,0.0)

Fully adjusted outcome model includes pre-baseline and baseline confounders: age, education, race, ethnicity, income, alcohol intake, sleeping duration, smoking status, CES-D depression score, HEI 2015 diet score, total physical activity intensity (MET-hrs), marital status, diabetes at baseline, and cancer at baseline. Additionally, pre-baseline intake of daily total protein and both pre-baseline and baseline versions of our outcome measurements were also adjusted.

S1 Table 8: Estimated levels of android and gynoid regional outcomes at end of follow-up after 3 years of hypothetical (categorical) protein intake interventions in post-menopausal women in the 1993 – 1998 Women's Health Initiative (WHI) observational study DXA sub-cohort.

Assigned Protein Intake Interventions (Baseline)	Android Adipose Tissue (kg)	Android Lean Soft Tissue mass (kg)	Gynoid Adipose Tissue (kg)	Gynoid Lean Soft Tissue mass (kg)

0	Natural Course (no intervention)	2.5 (2.4,2.6)	2.9 (2.9,3.0)	5.5 (5.4,5.6)	5.9 (5.8,5.9)
1	≤ 0.8g/kg/day (reference)	2.6 (2.5,2.7)	3.0 (2.9,3.0)	5.6 (5.6,5.8)	5.9 (5.8,5.9)
2	> 0.8 but ≤ 1g/kg/day	2.5 (2.4,2.6)	3.0 (2.9,3.0)	5.5 (5.4,5.7)	5.9 (5.8,6.0)
3	>1 but ≤ 1.2g/kg/day	2.4 (2.3,2.5)	2.9 (2.9,3.0)	5.4 (5.3,5.6)	5.8 (5.7,5.9)
4	> 1.2 but ≤ 1.5g/kg/day	2.4 (2.3,2.5)	2.9 (2.9,3.0)	5.4 (5.2,5,5)	5.8 (5.8,5.9)
5	> 1.5 g/kg/day	2.4 (2.2,2.5)	2.8 (2.7,2.9)	5.3 (5.1,5.5)	5.7 (5.6,5.9)
**Average Treatment Effects**					
Intervention 2 vs 1		-0.1 (-0.2,0.0)	0.0 (-0.1,0.0)	-0.1 (-0.3,0.0)	0.0 (-0.1,0.1)
Intervention 3 vs 1		-0.2 (-0.3,0.0)	-0.1 (-0.1,0.0)	-0.2 (-0.4,-0.1)	-0.1 (-0.1,0.0)
Intervention 4 vs 1		-0.2 (-0.3,-0.1)	-0.1 (-0.1,0.0)	-0.2 (-0.5,-0.1)	-0.1 (-0.1,0.0)
Intervention 5 vs 1		-0.2 (-0.4,-0.1)	-0.2 (-0.2,-0.1)	-0.3 (-0.6,-0.1)	-0.2 (-0.3,0.0)
Fully adjusted outcome model includes pre-baseline and baseline confounders: age, education, race, ethnicity, income, alcohol intake, sleeping duration, smoking status, CES-D depression score, HEI 2015 diet score, total physical activity intensity (MET-hrs), marital status, diabetes at baseline, and cancer at baseline. Additionally, pre-baseline intake of daily total protein and both pre-baseline and baseline versions of our outcome measurements were also adjusted.					

S1 Table 9: Mean total daily protein intake, total daily energy intake, and percentage of total daily energy intake coming from protein, grouped by different protein intake categories.

Protein Intake Group	N (%)[a]	Mean total daily protein intake (g)[b]	Mean total daily energy intake (kcals)[c]	Mean total daily energy intake (kJ)[c]	Mean percentage of total daily energy intake coming from protein (%)[d]
≤ 0.8g/kg/day	1862 (45.5)	47.0	1170.7	4898.2	16.2
> 0.8 but ≤ 1g/kg/day	799 (19.5)	62.7	1466.1	6134.2	17.6
>1 but ≤ 1.2g/kg/day	551 (13.5)	74.4	1708.9	7150.0	17.8
> 1.2 but ≤ 1.5g/kg/day	490 (12.0)	87.6	1946.8	8145.8	18.4
> 1.5 g/kg/day	389 (9.5)	126.1	2720.3	11381.7	19.0

[a] Count of participants in study per protein intake category group.
[b] Mean total daily protein intake (grams) refers to the average daily protein intake at baseline, calculated for each group.
[c] Mean total daily energy intake (kcals) or kJ refers to the average total energy intake at baseline, calculated for each group.
[d] To calculate the mean percentage of total daily energy intake from protein, each participant's protein intake (in grams) was multiplied by 4 kcal/g to obtain protein-derived calories. This value was then divided by the participant's total daily caloric intake and multiplied by 100 to yield the % of energy from protein. Participants were grouped into the predefined protein intake categories, and the mean percentage was then calculated within each group.

**S1 Table 10: Mean total daily protein intake and mean total daily protein intake per kilogram of bodyweight, stratified by categorical BMI.**			
Categorical BMI	N (%)[a]	Mean total daily protein intake (g)[b]	Mean total daily protein intake per kg bodyweight (g/kg)[c]
< 18.5	55 (1.2)	62.6 (33.0)	1.0 (0.5)
18.5 ≤ but < 24	1270 (27.8)	58.9 (29.4)	1.1 (0.5)
24 ≤ but < 30	1964 (43.1)	61.9 (30.8)	0.9 (0.5)
30 ≤ but < 35	824 (18.1)	66.6 (34.8)	0.8 (0.4)
≥ 35	449 (9.8)	71.9 (49.1)	0.7 (0.6)

[a] Count of participants in study per BMI range.
[b] Mean total daily protein (grams) intake refers to the average daily protein intake at baseline, calculated for each categorical BMI group.
[c] Mean total daily protein intake divided by participant's bodyweight in kilograms (g/kg), calculated for each categorical BMI group.

**S1 Table 11: Mean total energy intake at baseline, stratified by categorical baseline VAT quintiles.**			
VAT	N (%)	Mean Total Daily Energy Intake (kcal)[a]	Mean Total Daily Energy Intake (kJ)[a]
Q1 (mean 58.2; SD 20.8)	835 (20.0)	1402.5 (611.8)	5871.1 (2559.1)
Q2 (mean 110.7; SD 11.9)	836 (20.0)	1458.1 (683.6)	6102.8 (2860.2)
Q3 (mean 150.5; SD 11.8)	836 (20.0)	1464.9 (668.8)	6132.7 (2799.6)
Q4 (mean 194.8; SD 15.6)	836 (20.0)	1497.3 (764.1)	6265.6 (3197.7)
Q5 (mean 282.8; SD 55.0)	835 (20.0)	1598.9 (914.2)	6688.7 (3826.5)

[a] Mean total daily energy intake (kcals) or kJ refers to the average total energy intake at baseline, calculated for each quintile.

**S1 Table 12: Mean total energy intake at baseline, stratified by categorical baseline SAT quintiles.**			
SAT	N (%)	Mean Total Daily Energy Intake (kcal)[a]	Mean Total Daily Energy Intake (kJ)[a]
Q1 (mean 192.3; SD 41.4)	835 (20.0)	1450.2 (679.5)	6068.1 (2842.5)
Q2 (mean 285.4; SD 20.2)	836 (20.0)	1397.4 (626.6)	5847.1 (2622.7)
Q3 (mean 353.0; SD 20.1)	836 (20.0)	1428.6 (661.4)	5978.6 (2767.3)

Q4 (mean 424.3; SD 22.5)	836 (20.0)	1494.7 (698.0)	6254.4 (2921.6)
Q5 (mean 566.2; SD 91.3)	835 (20.0)	1696 (931.3)	7095.3 (3897.7)
[a] Mean total daily energy intake (kcals) or kJ refers to the average total energy intake at baseline, calculated for each quintile.			

**S1 Table 13: Mean total energy intake at baseline, stratified by categorical baseline total bodyfat percent quintiles.**			
Total Bodyfat (%)	N (%)	Mean Total Daily Energy Intake (kcal)[a]	Mean Total Daily Energy Intake (kJ)[a]
Q1 (mean 32.5; SD 4.6)	831 (20.0)	1451.6 (698.7)	6074.6 (2923.7)
Q2 (mean 40.2; SD 1.3)	831 (20.0)	1438.5 (667.7)	6018.1 (2794.1)
Q3 (mean 44.1; SD 1.0)	831 (20.0)	1499.6 (721.5)	6274.3 (3018.8)
Q4 (mean 47.7; SD 1.1)	831 (20.0)	1487.4 (739.2)	6223.7 (3093.4)
Q5 (mean 53.2; SD 2.7)	831 (20.0)	1543.3 (852.1)	6458.2 (3564.8)
[a] Mean total daily energy intake (kcals) or kJ refers to the average total energy intake at baseline, calculated for each quintile.			

**S1 Table 14: Mean total energy intake at baseline, stratified by categorical baseline total lean mass percent quintiles.**			
Total Lean Mass (%)	N (%)	Mean Total Daily Energy Intake (kcal)[a]	Mean Total Daily Energy Intake (kJ)[a]
Q1 (mean 44.2; SD 2.5)	831 (20.0)	1544.2 (848.1)	6462.0 (3548.9)
Q2 (mean 49.5; SD 1.1)	831 (20.0)	1505.5 (775.1)	6299.0 (3243.2)
Q3 (mean 52.9; SD 1.0)	831 (20.0)	1487.7 (679.5)	6225.0 (2842.5)
Q4 (mean 56.7; SD 1.3)	831 (20.0)	1444.5 (730.5)	6043.3 (3057.3)
Q5 (mean 64.2; SD 4.5)	831 (20.0)	1438.6 (642.6)	6018.5 (2688.4)
[a] Mean total daily energy intake (kcals) or kJ refers to the average total energy intake at baseline, calculated for each quintile.			

**S1 Table 15: Mean total energy intake at baseline, stratified by categorical baseline total bodyweight quintiles.**			
Total Bodyweight (kg)	N (%)	Mean Total Daily Energy Intake (kcal)[a]	Mean Total Daily Energy Intake (kJ)[a]
Q1 (mean 52.6; SD 4.1)	831 (20.0)	1451.7 (676.9)	6075.0 (2833.3)
Q2 (mean 61.5; 2.0)	831 (20.0)	1334.7 (651.7)	5587.3 (2727.3)

Q3 (mean 68.3; SD 2.0)	831 (20.0)	1461.7 (653.3)	6117.4 (2734.8)
Q4 (mean 76.4; SD 2.9)	831 (20.0)	1436.7 (586.4)	6010.4 (2454.3)
Q5 (mean 94.3; SD 11.1)	831 (20.0)	1628.2 (833.6)	6814.6 (3488.6)
[a] Mean total daily energy intake (kcals) or kJ refers to the average total energy intake at baseline, calculated for each quintile.			

S1 Table 16: Mean total energy intake at baseline, stratified by age group.

Age at screening	N (%)	Mean Total Daily Energy Intake (kcal)[a]	Mean Total Daily Energy Intake (kJ)[a]
Age < 65	2230 (53.4)	1530.2 (786.4)	6404.0 (3291.1)
Age ≥ 65	1948 (46.6)	1431.3 (675.1)	5988.6 (2823.1)
[a] Mean total daily energy intake (kcals) or kJ refers to the average total energy intake at baseline, calculated for each age group.			

Supplemental File S2

S2 Table 1: Estimated levels of Visceral Adipose Tissue (VAT), Subcutaneous Adipose Tissue (SAT), total body fat percentage, total lean soft tissue mass percentage, and total bodyweight at end of follow-up after 3 years of hypothetical protein intake interventions in post-menopausal women in the 1993 – 1998 Women's Health Initiative (WHI) observational study DXA sub-cohort.

Interventions		VAT (cm^2)	SAT (cm^2)	Total Body Fat (%)	Total Lean soft tissue mass (%)	Weight (kg)
0	Natural Course (reference, no intervention)	169.3 (165.8, 172.8)	367.5 (359.8, 375.2)	43.2 (42.8, 43.6)	53.9 (53.5, 54.3)	71.6 (70.5, 72.2)
1	≥ 0.8 g/kg/day	174.3 (171.1, 177.5)	372.1 (364.7, 379.5)	43.5 (43.1, 43.9)	53.6 (53.2, 54.0)	72.0 (71.1, 72.9)
2	≥ 1.0 g/kg/day	169.6 (165.8, 173.4)	364.6 (356.4, 372.9)	43.2 (42.8, 43.6)	53.9 (53.5, 54.2)	71.2 (70.3, 72.2)
3	≥ 1.2 g/kg/day	164.9 (160.0, 169.9)	357.1 (346.4, 367.8)	42.9 (42.4, 43.4)	54.1 (53.7, 54.6)	70.5 (69.4, 71.6)
4	≥ 1.5 g/kg/day	157.9 (150.7, 165.2)	345.9 (330.2, 361.6)	42.5 (41.8, 43.2)	54.5 (53.9, 55.2)	69.4 (68.0, 70.8)
**Average Treatment Effects**						
Intervention 1 vs 0		5.0 (2.6, 7.3)	4.6 (1.1, 8.1)	0.3 (0.1, 0.5)	-0.3 (-0.4, -0.1)	0.6 (0.2, 1.0)
Intervention 2 vs 0		0.3 (-2.0, 2.6)	-2.9 (-7.7, 1.9)	0.0 (-0.2, 0.3)	0.0 (-0.3, 0.2)	-0.1 (-0.6, 0.4)
Intervention 3 vs 0		-4.4 (-7.7, -1.1)	-10.4 (-18.5, -2.2)	-0.3 (-0.7, 0.1)	0.3 (-0.2, 0.6)	-0.8 (-1.6, -0.1)
Intervention 4 vs 0		-11.4 (-17.0, -5.8)	-21.6 (-35.5, -7.7)	-0.7 (-1.4, 0.0)	0.7 (0.0, 1.3)	-2.0 (-3.1, -0.8)

Fully adjusted outcome model includes pre-baseline and baseline confounders: age, education, race, ethnicity, income, alcohol intake, sleeping duration, smoking status, CES-D depression score, HEI 2015 diet score, total energy intake, total physical activity intensity (MET-hrs), marital status, diabetes at baseline, and cancer at baseline. Additionally, pre-baseline intake of daily total protein and both pre-baseline and baseline versions of our outcome measurements were also adjusted.

S2 Table 2: Estimated levels of trunk adipose tissue and lean Soft Tissue mass at end of follow-up after 3 years of hypothetical protein intake interventions in post-menopausal women in the 1993 – 1998 Women's Health Initiative (WHI) observational study DXA sub-cohort.

Interventions		Trunk Adipose Tissue (kg)	Trunk Lean Soft Tissue mass (kg)
0	Natural Course (reference, no intervention)	14.8 (14.4, 15.1)	19.7 (19.5, 19.9)
1	≥ 0.8 g/kg/day	15.1 (14.7, 15.4)	19.8 (19.6, 19.9)
2	≥ 1.0 g/kg/day	14.7 (14.4, 15.1)	19.6 (19.5, 19.8)
3	≥ 1.2 g/kg/day	14.4 (14.0, 14.9)	19.5 (19.3, 19.7)
4	≥ 1.5 g/kg/day	13.9 (13.3, 14.6)	19.3 (19.1, 19.5)
**Average Treatment Effects**			
Intervention 1 vs 0		0.3 (0.1, 0.5)	0.1 (0.0, 0.2)
Intervention 2 vs 0		0.0 (-0.2, 0.2)	0.0 (-0.1, 0.0)
Intervention 3 vs 0		-0.3 (-0.7, 0.0)	-0.2 (-0.2, -0.1)
Intervention 4 vs 0		-0.8 (-1.4, -0.3)	-0.3 (-0.5, -0.2)

Fully adjusted outcome model includes pre-baseline and baseline confounders: age, education, race, ethnicity, income, alcohol intake, sleeping duration, smoking status, CES-D depression score, HEI 2015 diet score, total energy intake, total physical activity intensity (MET-hrs), marital status, diabetes at baseline, and cancer at baseline. Additionally, pre-baseline intake of daily total protein and both pre-baseline and baseline versions of our outcome measurements were also adjusted.

S2 Table 3: Estimated levels of appendicular regional outcomes at end of follow-up after 3 years of hypothetical protein intake interventions in post-menopausal women in the 1993 – 1998 Women's Health Initiative (WHI) observational study DXA sub-cohort.

Interventions		Total Leg Adipose Tissue (kg)	Total Leg Lean Soft Tissue mass (kg)	Total Arm Adipose Tissue (kg)	Total Arm Lean Soft Tissue mass (kg)
0	Natural Course (reference)	11.7 (11.5, 12.0)	11.3 (11.2, 11.4)	4.5 (4.4, 4.6)	3.4 (3.4, 3.5)
1	≥ 0.8 g/kg/day	11.9 (11.6, 12.1)	11.4 (11.2, 11.5)	4.6 (4.4, 4.7)	3.5 (3.4, 3.5)
2	≥ 1.0 g/kg/day	11.8 (11.5, 12.0)	11.3 (11.2, 11.4)	4.4 (4.3, 4.5)	3.4 (3.4, 3.5)
3	≥ 1.2 g/kg/day	11.7 (11.4, 11.9)	11.2 (11.1, 11.3)	4.1 (4.0, 4.3)	3.4 (3.3, 3.4)
4	≥ 1.5 g/kg/day	11.5 (11.1, 11.9)	11.1 (10.9, 11.2)	3.8 (3.5, 4.1)	3.3 (3.2, 3.4)
**Average Treatment Effects**					
Intervention 1 vs 0		0.1 (0.0, 0.3)	0.0 (0.0, 0.1)	0.1 (0.0, 0.2)	0.0 (0.0, 0.1)
Intervention 2 vs 0		0.0 (-0.1, 0.2)	-0.1 (-0.1, 0.0)	-0.1 (-0.2, 0.0)	0.0 (-0.1, 0.0)
Intervention 3 vs 0		-0.1 (-0.3, 0.2)	-0.1 (-0.2, 0.0)	-0.3 (-0.4, -0.1)	-0.1 (-0.1, 0.0)
Intervention 4 vs 0		-0.2 (-0.6, 0.2)	-0.3 (-0.4, -0.1)	-0.7 (-0.9, -0.3)	-0.2 (-0.2, -0.1)

Fully adjusted outcome model includes pre-baseline and baseline confounders: age, education, race, ethnicity, income, alcohol intake, sleeping duration, smoking status, CES-D depression score, HEI 2015 diet score, total energy intake, total physical activity intensity (MET-hrs), marital status, diabetes at baseline, and cancer at baseline. Additionally, pre-baseline intake of daily total protein and both pre-baseline and baseline versions of our outcome measurements were also adjusted.

S2 Table 4: Estimated levels of android and gynoid regional outcomes at end of follow-up after 3 years of hypothetical protein intake interventions in post-menopausal women in the 1993 – 1998 Women's Health Initiative (WHI) observational study DXA sub-cohort.

Interventions		Android Adipose Tissue (kg)	Android Lean Soft Tissue mass (kg)	Gynoid Adipose Tissue (kg)	Gynoid Lean Soft Tissue mass (kg)
0	Natural Course (reference)	2.5 (2.4, 2.6)	2.9 (2.9, 3.0)	5.5 (5.5, 5.6)	5.9 (5.8, 5.9)
1	≥ 0.8 g/kg/day	2.6 (2.5, 2.6)	3.0 (2.9, 3.0)	5.6 (5.5, 5.7)	5.9 (5.8, 5.9)
2	≥ 1.0 g/kg/day	2.5 (2.4, 2.6)	2.9 (2.9, 3.0)	5.5 (5.4, 5.6)	5.9 (5.8, 6.0)
3	≥ 1.2 g/kg/day	2.4 (2.4, 2.5)	2.9 (2.8, 2.9)	5.4 (5.3, 5.5)	5.8 (5.8, 5.9)
4	≥ 1.5 g/kg/day	2.4 (2.2, 2.5)	2.8 (2.7, 2.9)	5.3 (5.1, 5.5)	5.8 (5.7, 5.8)
**Average Treatment Effects**					
Intervention 1 vs 0		0.1 (0.0, 0.1)	0.0 (0.0, 0.1)	0.1 (0.1, 0.2)	0.0 (0.0, 0.1)

Intervention 2 vs 0	0.0 (-0.1, 0.0)	0.0 (0.0, 0.0)	0.0 (0.0, 0.1)	0.0 (0.0, 0.0)
Intervention 3 vs 0	-0.1 (-0.2, 0.0)	0.0 (-0.1, 0.0)	-0.1 (-0.2, 0.0)	-0.1 (-0.1, 0.0)
Intervention 4 vs 0	-0.2 (-0.3, -0.1)	-0.1 (-0.2, -0.1)	-0.2 (-0.4, -0.1)	-0.1 (-0.2, -0.1)

Fully adjusted outcome model includes pre-baseline and baseline confounders: age, education, race, ethnicity, income, alcohol intake, sleeping duration, smoking status, CES-D depression score, HEI 2015 diet score, total energy intake, total physical activity intensity (MET-hrs), marital status, diabetes at baseline, and cancer at baseline. Additionally, pre-baseline intake of daily total protein and both pre-baseline and baseline versions of our outcome measurements were also adjusted.

Supplemental File S3

S3 Figure 1. Estimated levels of Visceral Adipose Tissue (VAT) at end of follow-up after 3 years of hypothetical protein intake interventions in post-menopausal women in the 1993 – 1998 Women's Health Initiative (WHI) observational study DXA sub-cohort.

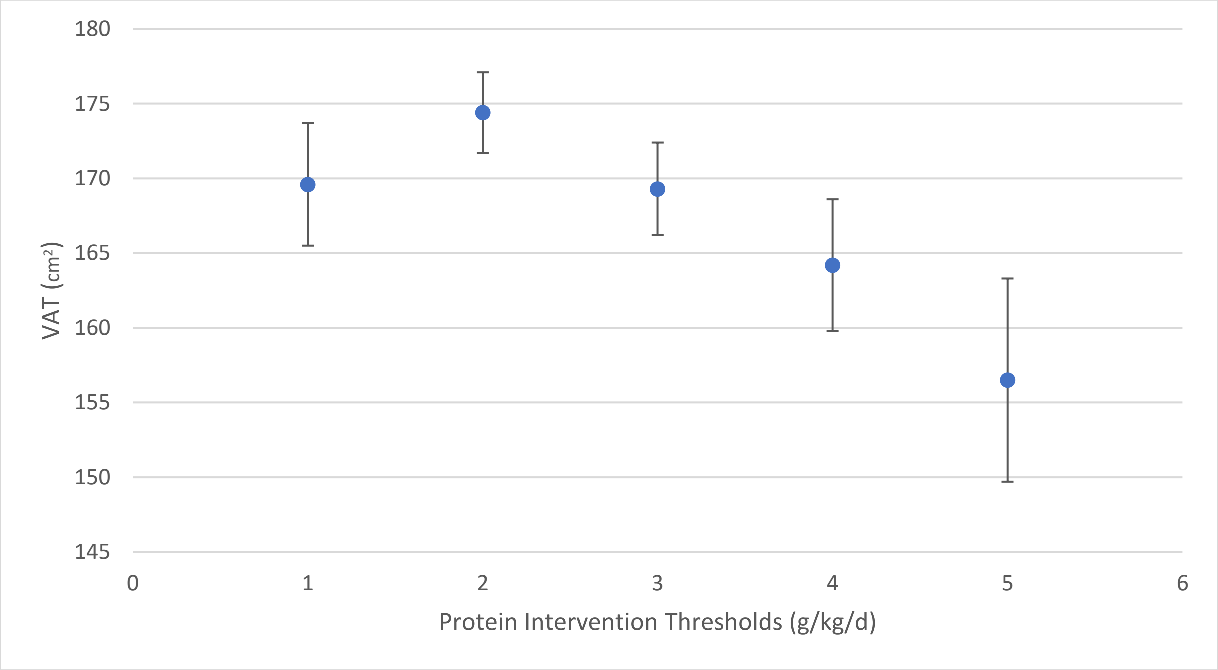

S3 Figure 2. Estimated levels of Subcutaneous Adipose Tissue (SAT) at end of follow-up after 3 years of hypothetical protein intake interventions in post-menopausal women in the 1993 – 1998 Women's Health Initiative (WHI) observational study DXA sub-cohort.

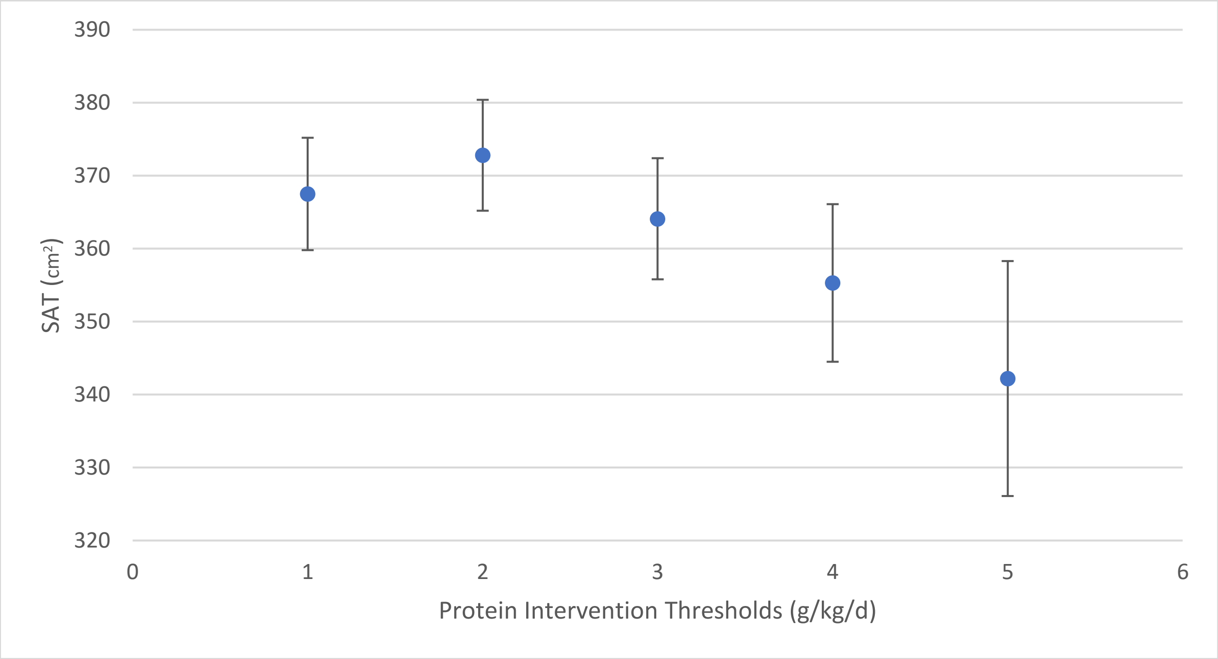

S3 Figure 3. Estimated total bodyfat (%) level at end of follow-up after 3 years of hypothetical protein intake interventions in post-menopausal women in the 1993 – 1998 Women's Health Initiative (WHI) observational study DXA sub-cohort.

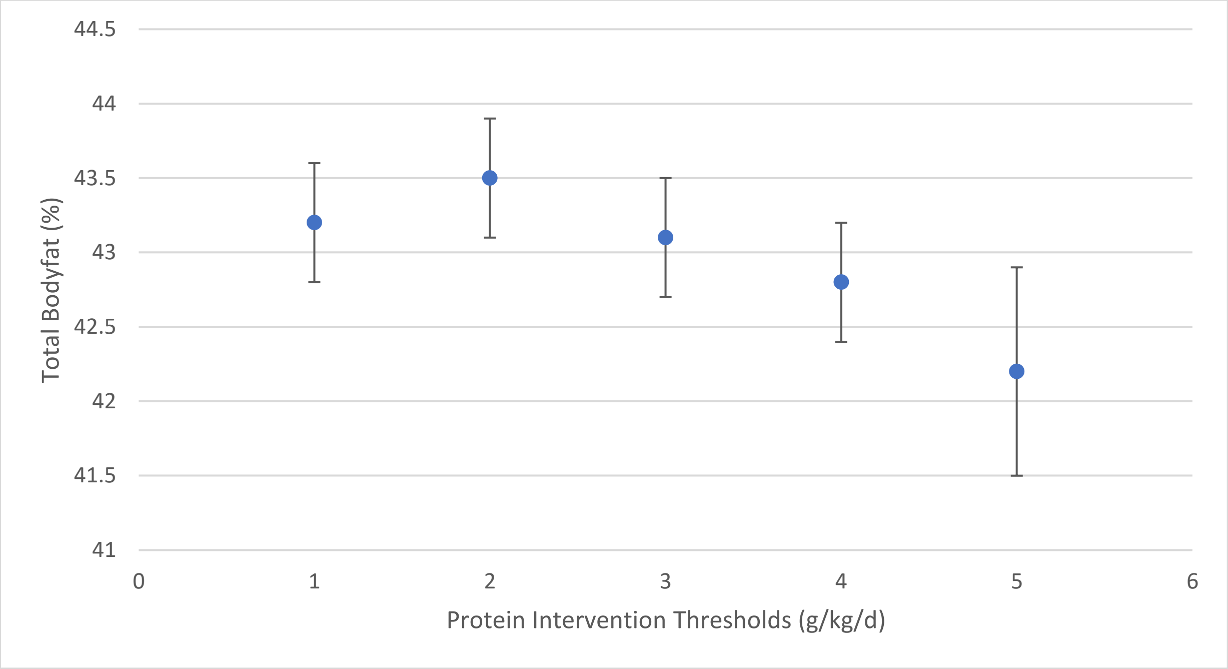

S3 Figure 4. Estimated total lean soft tissue (%) level at end of follow-up after 3 years of hypothetical protein intake interventions in post-menopausal women in the 1993 – 1998 Women's Health Initiative (WHI) observational study DXA sub-cohort.

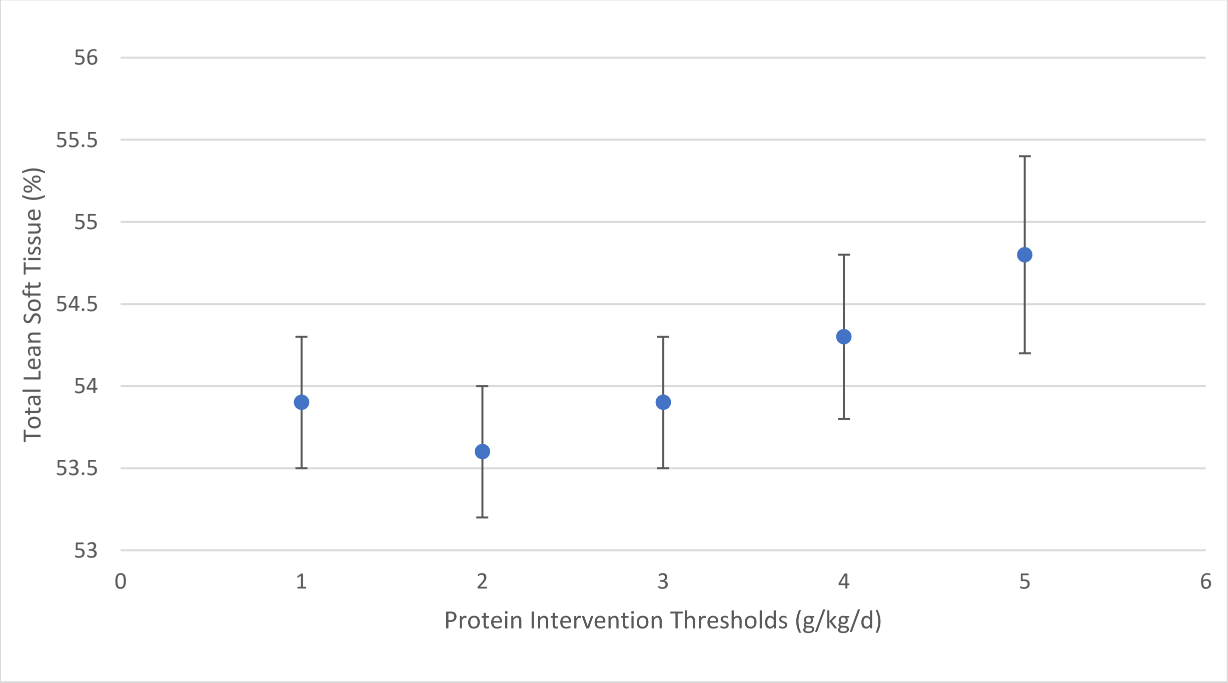

S3 Figure 5. Estimated total bodyweight (kg) at end of follow-up after 3 years of hypothetical protein intake interventions in post-menopausal women in the 1993 – 1998 Women's Health Initiative (WHI) observational study DXA sub-cohort.

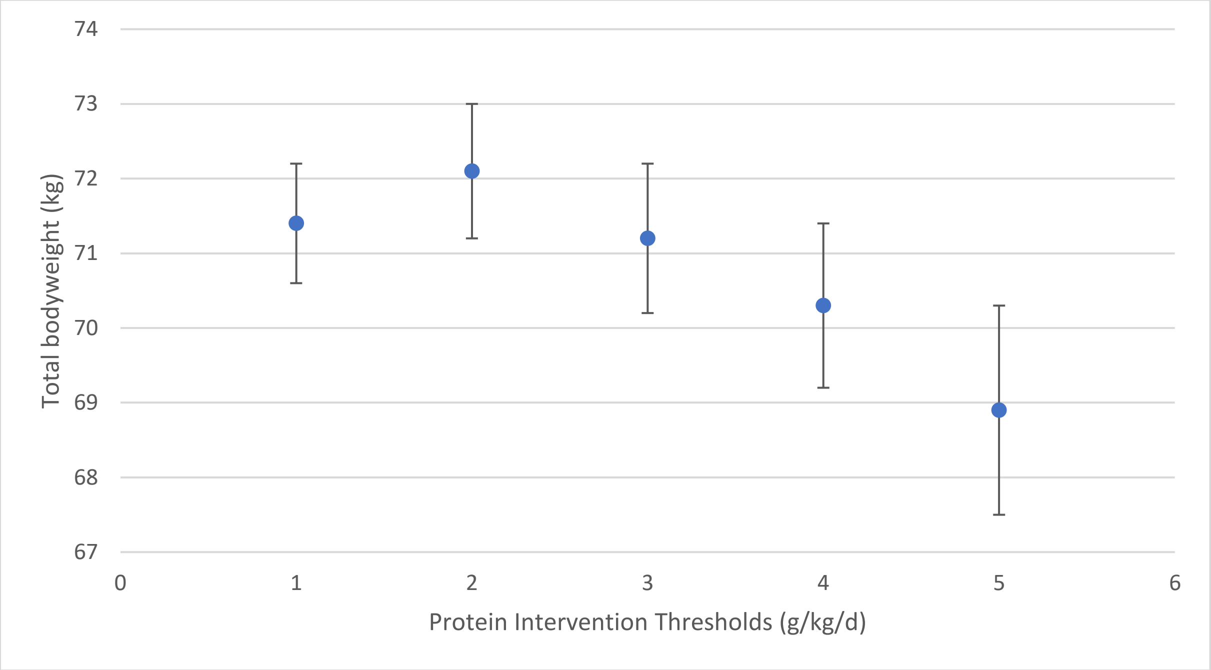

Supplemental File Assumptions Clarifications

Exchangeability

Imagine we are doing a clinical experiment and we want to know if drinking protein shakes helps weight loss. The best way to figure this out would be to randomly split participants into two groups where one group gets the shakes while the other doesn't (making sure everything else about them is basically the same). This is the core concept of exchangeability. It means the two groups are so similar that any difference in the outcome (like weight loss) is due to the protein shakes, not anything else. Now, in our study, we didn't run a real experiment because we used existing data, like one would see in surveys or health data. As such, we can't randomly assign people to a group of protein shake drinkers and non-drinkers. Instead, we try to adjust for differences between participants using information we already know about them (e.g. the participants' age, activity level, or education level). This is our study's assumption of conditional exchangeability. It means: 'As long as we've accounted for all the important covariates that could affect the results, and assume there are no other important covariates other than the ones we have included in our study, then our comparison is still fair.'

Positivity

In this same experiment, every participant also has some chance of being assigned to either the protein shakes group or the no-shake group (no participant is automatically excluded from either option just because of something like age or alcohol intake level). This is the core idea of positivity. It means that for every combination of characteristics (e.g. age, alcohol intake, or marriage status), there is a real chance to observe a participant in both the treatment and comparison groups.

Again, in our current study, we are not conducting an experiment but rather relying on statistical analyses of existing data. That means there are no assigned groups, and instead the data reflects what participants were already doing. For the analysis to work well, the assumption of positivity must still hold: it must be true that within every group defined by relevant characteristics (for example, older adults with low physical activity), there are at least some participants across the full range of protein intakes being studied. If certain groups only ever appear with too low or high protein intake, it becomes impossible to estimate what would happen if they had a different intake and the model could break down.

SUTVA

SUTVA stands for the Stable Unit Treatment Value Assumption. In short, it's a rule that helps make sure a study is actually measuring what it's supposed to measure.

The first component is **no interference.** This means one participant's outcome should not be affected by another participant in the study. A violation of this component could be a healthy eating class-room treatment where due to peer pressure some individuals also finish eating their healthier meal trays (if that is a treatment they were assigned) when they may not have if they were eating on their own.

The second component entails **well-defined treatments**. This means the treatment being studied has to be clearly defined and consistent across everyone who receives it. If two people are said to be on a “high-protein” diet, then it should actually mean the same thing in terms of amount, source, and timing. If not, it’s like comparing apples and oranges.

Together, these two components maybe an afterthought, especially in experimental settings where the researchers will actively define treatment specification and guidance. However, in observational data, there is no control on how an exposure is defined or the setting it’s given – therefore, we need to make this assumption in order for our inferences to be valid. In some literature, SUTVA is also often defined in line with the consistency assumption, which says that the outcome someone gets under a specific treatment is exactly what would be expected if that treatment were actually applied in real life.

No model mis-specification

Even when all the key assumptions are satisfied, it’s important to remember that we’re still relying on statistical models to estimate the relationship between our exposure (protein intake), confounders, and the outcome (such as a body composition measure). This means we must also assume that the way we’ve specified the model (such as how we’ve included covariates) accurately reflects their true relationship with the outcome. For instance, if a covariate actually has a non-linear relationship with the outcome, but we include it in the model as a simple linear term (without, say, squaring it or using another transformation), our model may still produce biased or incorrect estimates, even though we technically adjusted for that covariate. Assuming no model misspecification is an important additional assumption, there’s no definitive way to know the ‘correct’ functional form of the relationship between covariates and the outcome unless one had access to the true underlying rules that govern how those variables interact.

Supplemental File: Power analysis

When planning a study/analysis with g-formula (and any other g-methods), determining the required sample size for a given level of statistical power is challenging because of the effect of hypothetical interventions on the variance of the estimated causal means. Sample size calculations are estimated akin to a randomized trial, however there is evidence from simulation studies that similar sample sizes in real v. emulated trials have different power, due to the "synthetic" nature of the data being compared (emulated tend to have lower statistical power).[1]

In this study, the primary outcome will be changes in lean soft tissue (%), body fat (%), total bodyweight (kg), visceral adipose tissue (cm^2), and subcutaneous adipose tissue (cm^2) over 3 years of follow-up. Ultimately, the power analysis assumes that the g-formula will create a conditionally exchangeable (ignorable) pseudo-population for comparison, and thus we expect to be comparing four groups of ~4,681 participants per protein level intake (e.g., 4,681 ≥0.8 g/kg/d v. 4,681 ≥1.0 g/kg/d v. 4,681 ≥1.2 g/kg/d v. 4,681 ≥1.5 g/kg/d).

- Our preliminary data showed that the mean ± standard deviation of lean soft tissue (%) is 54.2 ± 7.2 %. The mean ± standard deviation of body fat (%) is 42.8 ± 7.5 %. The mean ± standard deviation of bodyweight (kg) is 70 ± 15. The mean ± standard deviation of SAT and VAT across time points are 359 ±135 cm^2 and 154±82 cm^2 respectively.
- Lean soft tissue (%) power: Assuming an n=9362 will enter this two-treatment parallel-design study (emulated). The probability is 90 percent that the study will detect a treatment difference at a two-sided 0.05 significance level if the true difference between treatments is .48%. This assumes that the standard deviation of the response variable is 7.2%. Thus, even with the expected inflated variance we are highly powered to detect a small difference in lean soft tissue (%) over time.
- Body fat (%) power: Assuming an n=9362 will enter this two-treatment parallel-design study (emulated). The probability is 90 percent that the study will detect a treatment difference at a two-sided 0.05 significance level if the true difference between treatments is 0.50%. This assumes that the standard deviation of the response variable is 7.5%. Thus, even with the expected inflated variance we are highly powered to detect a small difference in body fat (%) over time.
- Total bodyweight (%) power: Assuming an n=9362 will enter this two-treatment parallel-design study (emulated). The probability is 90 percent that the study will detect a treatment difference at a two-sided 0.05 significance level if the true difference between treatments is 1.0 kg. This assumes that the standard deviation of the response variable is 15kg. Thus, even with the expected inflated variance we are highly powered to detect a small difference in total bodyweight (kg) over time.
- VAT power: Assuming an n=9362 will enter this two-treatment parallel-design study (emulated). The probability is 90 percent that the study will detect a treatment difference at a two-sided 0.05 significance level if the true difference between treatments is units 5.5 (cm^2). This assumes that the standard deviation of the response variable is 82 (VAT). Thus, even

with the expected inflated variance we are highly powered to detect a small difference in VAT over time.

- SAT power: Assuming an n=9362 will enter this two-treatment parallel-design study (emulated). The probability is 90 percent that the study will detect a treatment difference at a two-sided 0.05 significance level if the true difference between treatments is units 9.1 (cm^2). This assumes that the standard deviation of the response variable is 135 (SAT). Thus, even with the expected inflated variance we are highly powered to detect a small difference in SAT over time.

Overall, we will be cautious to interpret estimate effects as clinically significant even though they may be statistically different. Of note, clinical/expert consensus attributes change of weight/adipose tissue amounts ~5% as clinically significant.

Supplemental File: Target Trial Emulation Addendum

The target trial emulation framework is a recently developed approach designed to enhance the rigor of statistical estimates from observational studies, gradually enabling researchers to embrace the idea that the associations identified are indeed causal.[2] The process is essentially a conceptual framework that emphasizes explicitly defining each component of the study, mirroring the elements of a randomized clinical trial (RCT).[3,4] RCTs are often considered the gold standard for generating causal estimates because they create comparable groups, allowing differences in outcomes to be attributed to the treatment rather than to prognostic differences between groups.

The target trial is essentially a two-step process.[3] The process begins by articulating the causal question and explicitly defining each component of the observational study in the framework of a hypothetical, idealized randomized trial. This includes specifying the eligibility criteria, treatment strategies, treatment assignment, follow-up period, outcomes, and causal contrasts.

Eligibility criteria are based on participants' characteristics and should be determined using baseline values only. Formulating the treatment strategies and assignment follows, where it's important to define the number of treatment options, the duration of adherence, and whether the treatment is assigned through a probability-based approach (e.g., 60% receive a higher dose, 40% a minimum dose) or a static approach (same dose throughout the study). In actual randomized clinical trials (RCTs), treatment is assigned via active randomization, but in observational studies, this is emulated by adjusting for a carefully selected set of confounders, ideally identified through domain expertise and causal directed acyclic graphs.[5]

The next step is defining the outcome, and the measurements used to quantify it, ensuring these are clearly stated and backed by tools that verify their validity and reliability. Then, follow-up is defined, typically extending from the study's baseline until the outcome occurs, a censoring event happens, death (or other competing events) occurs, or the administrative end of follow-up is reached.[6]

Defining the causal contrast of interest follows. In RCTs, the main contrast is usually the intent-to-treat effect, and with observational data, this approach can be applied if treatment assignment data is available. However, observational studies often target the per-protocol effect, which captures the effect observed under full adherence to the assigned treatment strategy.

Finally, selecting the appropriate statistical methodology is crucial for accurately estimating effects. Conventional methods can adjust for baseline covariates in non-time-varying settings, providing conditional effects. In contrast, g-methods—such as inverse probability weighted marginal structural models, the parametric g-formula, and g-estimation of structural nested models—allow for identification and estimation of effects under less restrictive assumptions, offering population-level contrast estimates that align well with comparisons of all treated versus all untreated individuals.[5,7,8]

After specifying the target trial, the second step of the target trial emulation is now starting the emulation process by choosing the appropriate estimation method and performing statistical analyses with them, such as one of the g-methods. The estimation model of choice in this paper, given its ability to efficiently account for multi-level treatment methods, is the parametric g-formula.[5] In short, parametric g-formula, in non-time-varying high-dimensional data settings, utilizes the concept of estimating a standardized mean Y written as $\int E[Y|A = a, C = 0, L = l] * dF_L(l)$ where $F_L(\cdot)$ is the joint cumulative distribution function (CDF) of random

variables in *L*, the vector of a list of baseline and pre-baseline confounding covariates unaffected by the treatment, where we average over the observed values of *L* to then estimate this integral. In this integral, *A* is the value of treatment strategy assigned into the dataset during the estimation process while *C* denotes censoring status.

In our observational data emulation setting, a frequently discussed concept is the natural course intervention, also known as no-intervention.[9] The causal contrast often involves the average treatment effect, comparing a treated population to an untreated population. However, in public health contexts, this type of contrast is increasingly recognized as unrealistic since it is typically impossible to ensure that all individuals in a population are fully unexposed, raising concerns about the validity of the estimates.[10,11]

The natural course or no-intervention approach represents a factual summary of the outcome under conditions that naturally occurred in the sample, where no intervention is applied, and treatment values remain unchanged. Plugging the fitted model with unchanged values here can and thus using the estimates as part of the control intervention strategy can offer insights into the effects of specific treatments outlined in the target trial emulation, showing how they might perform relative to real-world outcomes observed without intervention, rather than relying solely on a purely hypothetical comparison between fully treated and fully untreated scenarios.[9]

References

1. Austin PC, Schuster T, Platt RW. Statistical power in parallel group point exposure studies with time-to-event outcomes: an empirical comparison of the performance of randomized controlled trials and the inverse probability of treatment weighting (IPTW) approach. *BMC Med Res Methodol*. 2015;15(1):87. doi:10.1186/s12874-015-0081-3

2. Hernán MA, Robins JM. Using Big Data to Emulate a Target Trial When a Randomized Trial Is Not Available. *Am J Epidemiol*. 2016;183(8):758-764. doi:10.1093/aje/kwv254

3. Hernán MA, Wang W, Leaf DE. Target Trial Emulation: A Framework for Causal Inference From Observational Data. *JAMA*. 2022;328(24):2446-2447. doi:10.1001/jama.2022.21383

4. Matthews AA, Danaei G, Islam N, Kurth T. Target trial emulation: applying principles of randomised trials to observational studies. *BMJ*. 2022;378:e071108. doi:10.1136/bmj-2022-071108

5. Avenue 677 Huntington, Boston, Ma 02115. Causal Inference: What If (the book). Miguel Hernan's Faculty Website. October 19, 2012. Accessed July 14, 2024. https://www.hsph.harvard.edu/miguel-hernan/causal-inference-book/

6. Young JG, Stensrud MJ, Tchetgen Tchetgen EJ, Hernán MA. A causal framework for classical statistical estimands in failure-time settings with competing events. *Stat Med*. 2020;39(8):1199-1236. doi:10.1002/sim.8471

7. Hernán MA, Alonso A, Logan R, et al. Observational studies analyzed like randomized experiments: an application to postmenopausal hormone therapy and coronary heart disease. *Epidemiol Camb Mass*. 2008;19(6):766-779. doi:10.1097/EDE.0b013e3181875e61

8. Naimi AI, Cole SR, Kennedy EH. An introduction to g methods. *Int J Epidemiol*. 2017;46(2):756-762. doi:10.1093/ije/dyw323

9. Rudolph JE, Cartus A, Bodnar LM, Schisterman EF, Naimi AI. The Role of the Natural Course in Causal Analysis. *Am J Epidemiol*. 2021;191(2):341. doi:10.1093/aje/kwab248

10. Heckman JJ, Vytlacil E. Policy-Relevant Treatment Effects. *Am Econ Rev*. 2001;91(2):107-111. doi:10.1257/aer.91.2.107

11. Westreich D. From Patients to Policy: Population Intervention Effects in Epidemiology. *Epidemiol Camb Mass*. 2017;28(4):525. doi:10.1097/EDE.0000000000000648
