# Supplementary material for: Estimating the effect of hypothetical dietary protein interventions on changes in body composition of postmenopausal women over 3 years using data from the Women’s Health Initiative (WHI) Study: an emulated target trial
Source: Int J Obes (Lond). 2026 Jan 9;50(3):609–17. doi: 10.1038/s41366-025-01978-0 (PMC12965867; doi:10.1038/s41366-025-01978-0)
Supplement: Supplementary file 2 — Supplementary information [file 41366_2025_1978_MOESM2_ESM.docx]

| **S1 Table 10: Mean total daily protein intake and mean total daily protein intake per kilogram of bodyweight, stratified by categorical BMI.** | | | |
| --- | --- | --- | --- |
| Categorical BMI | N (%)^a^ | Mean total daily protein intake (g)^b^ | Mean total daily protein intake per kg bodyweight (g/kg)^c^ |
| < 18.5 | 55 (1.2) | 62.6 (33.0) | 1.0 (0.5) |
| 18.5 ≤ but < 24 | 1270 (27.8) | 58.9 (29.4) | 1.1 (0.5) |
| 24 ≤ but < 30 | 1964 (43.1) | 61.9 (30.8) | 0.9 (0.5) |
| 30 ≤ but < 35 | 824 (18.1) | 66.6 (34.8) | 0.8 (0.4) |
| ≥ 35 | 449 (9.8) | 71.9 (49.1) | 0.7 (0.6) |
| ^a^ Count of participants in study per BMI range.  ^b^ Mean total daily protein (grams) intake refers to the average daily protein intake at baseline, calculated for each categorical BMI group.  ^c^ Mean total daily protein intake divided by participant’s bodyweight in kilograms (g/kg), calculated for each categorical BMI group. | | | |

| **S1 Table 11: Mean total energy intake at baseline, stratified by categorical baseline VAT quintiles.** | | | |
| --- | --- | --- | --- |
| VAT | N (%) | Mean Total Daily Energy Intake (kcal)^a^ | Mean Total Daily Energy Intake (kJ)^a^ |
| Q1  (mean 58.2; SD 20.8) | 835 (20.0) | 1402.5 (611.8) | 5871.1 (2559.1) |
| Q2  (mean 110.7; SD 11.9) | 836 (20.0) | 1458.1 (683.6) | 6102.8 (2860.2) |
| Q3  (mean 150.5; SD 11.8) | 836 (20.0) | 1464.9 (668.8) | 6132.7 (2799.6) |
| Q4  (mean 194.8; SD 15.6) | 836 (20.0) | 1497.3 (764.1) | 6265.6 (3197.7) |
| Q5  (mean 282.8; SD 55.0) | 835 (20.0) | 1598.9 (914.2) | 6688.7 (3826.5) |
| ^a^ Mean total daily energy intake (kcals) or kJ refers to the average total energy intake at baseline, calculated for each quintile. | | | |

| **S1 Table 12: Mean total energy intake at baseline, stratified by categorical baseline SAT quintiles.** | | | |
| --- | --- | --- | --- |
| SAT | N (%) | Mean Total Daily Energy Intake (kcal)^a^ | Mean Total Daily Energy Intake (kJ)^a^ |
| Q1  (mean 192.3; SD 41.4) | 835 (20.0) | 1450.2 (679.5) | 6068.1 (2842.5) |
| Q2  (mean 285.4; SD 20.2) | 836 (20.0) | 1397.4 (626.6) | 5847.1 (2622.7) |
| Q3  (mean 353.0; SD 20.1) | 836 (20.0) | 1428.6 (661.4) | 5978.6 (2767.3) |
| Q4  (mean 424.3; SD 22.5) | 836 (20.0) | 1494.7 (698.0) | 6254.4 (2921.6) |
| Q5  (mean 566.2; SD 91.3) | 835 (20.0) | 1696 (931.3) | 7095.3 (3897.7) |
| ^a^ Mean total daily energy intake (kcals) or kJ refers to the average total energy intake at baseline, calculated for each quintile. | | | |

| **S1 Table 13: Mean total energy intake at baseline, stratified by categorical baseline total bodyfat percent quintiles.** | | | |
| --- | --- | --- | --- |
| Total Bodyfat (%) | N (%) | Mean Total Daily Energy Intake (kcal)^a^ | Mean Total Daily Energy Intake (kJ)^a^ |
| Q1 (mean 32.5; SD 4.6) | 831 (20.0) | 1451.6 (698.7) | 6074.6 (2923.7) |
| Q2 (mean 40.2; SD 1.3) | 831 (20.0) | 1438.5 (667.7) | 6018.1 (2794.1) |
| Q3 (mean 44.1; SD 1.0) | 831 (20.0) | 1499.6 (721.5) | 6274.3 (3018.8) |
| Q4 (mean 47.7; SD 1.1) | 831 (20.0) | 1487.4 (739.2) | 6223.7 (3093.4) |
| Q5 (mean 53.2; SD 2.7) | 831 (20.0) | 1543.3 (852.1) | 6458.2 (3564.8) |
| ^a^ Mean total daily energy intake (kcals) or kJ refers to the average total energy intake at baseline, calculated for each quintile. | | | |

| **S1 Table 14: Mean total energy intake at baseline, stratified by categorical baseline total lean mass percent quintiles.** | | | |
| --- | --- | --- | --- |
| Total Lean Mass (%) | N (%) | Mean Total Daily Energy Intake (kcal)^a^ | Mean Total Daily Energy Intake (kJ)^a^ |
| Q1 (mean 44.2; SD 2.5) | 831 (20.0) | 1544.2 (848.1) | 6462.0 (3548.9) |
| Q2 (mean 49.5; SD 1.1) | 831 (20.0) | 1505.5 (775.1) | 6299.0 (3243.2) |
| Q3 (mean 52.9; SD 1.0) | 831 (20.0) | 1487.7 (679.5) | 6225.0 (2842.5) |
| Q4 (mean 56.7; SD 1.3) | 831 (20.0) | 1444.5 (730.5) | 6043.3 (3057.3) |
| Q5 (mean 64.2; SD 4.5) | 831 (20.0) | 1438.6 (642.6) | 6018.5 (2688.4) |
| ^a^ Mean total daily energy intake (kcals) or kJ refers to the average total energy intake at baseline, calculated for each quintile. | | | |

| **S1 Table 15: Mean total energy intake at baseline, stratified by categorical baseline total bodyweight quintiles.** | | | |
| --- | --- | --- | --- |
| Total Bodyweight (kg) | N (%) | Mean Total Daily Energy Intake (kcal)^a^ | Mean Total Daily Energy Intake (kJ)^a^ |
| Q1 (mean 52.6; SD 4.1) | 831 (20.0) | 1451.7 (676.9) | 6075.0 (2833.3) |
| Q2 (mean 61.5; 2.0) | 831 (20.0) | 1334.7 (651.7) | 5587.3 (2727.3) |
| Q3 (mean 68.3; SD 2.0) | 831 (20.0) | 1461.7 (653.3) | 6117.4 (2734.8) |
| Q4 (mean 76.4; SD 2.9) | 831 (20.0) | 1436.7 (586.4) | 6010.4 (2454.3) |
| Q5 (mean 94.3; SD 11.1) | 831 (20.0) | 1628.2 (833.6) | 6814.6 (3488.6) |
| ^a^ Mean total daily energy intake (kcals) or kJ refers to the average total energy intake at baseline, calculated for each quintile. | | | |

| **S1 Table 16: Mean total energy intake at baseline, stratified by age group.** | | | |
| --- | --- | --- | --- |
| Age at screening | N (%) | Mean Total Daily Energy Intake (kcal)^a^ | Mean Total Daily Energy Intake (kJ)^a^ |
| Age < 65 | 2230 (53.4) | 1530.2 (786.4) | 6404.0 (3291.1) |
| Age ≥ 65 | 1948 (46.6) | 1431.3 (675.1) | 5988.6 (2823.1) |
| ^a^ Mean total daily energy intake (kcals) or kJ refers to the average total energy intake at baseline, calculated for each age group. | | | |
